# Supplementary material for: Getting Recovery Right After Neck Dissection (GRRAND-F): mixed-methods feasibility study to design a pragmatic randomised controlled trial protocol
Source: BMJ Open. 2021 Jun 21;11(6):e045741. doi: 10.1136/bmjopen-2020-045741 (PMC8217923; doi:10.1136/bmjopen-2020-045741)
Supplement: Supplementary data [file bmjopen-2020-045741supp001.pdf]

Sponsor Logo

CONSENT FORM (GRRAND-F STUDY)

GRRAND-F Getting Recovery Right After Neck Dissection

LOCAL TRUST LOGO

Name of Local Principal Investigator:___________________________

Screening ID: | G | D | S | — | | | — | | | |

If you agree, please initial

1. I confirm that I have read and understood the Information Leaflet dated 10 June 2020 Version 4.0. I have had the opportunity to consider the information, ask questions and have had these answered satisfactorily.	
2. I understand that my participation is voluntary and that I am free to withdraw at any time without giving any reason, and without my medical care or legal rights being affected.	
3. I understand that relevant sections of my medical notes and data collected during the study may be looked at by individuals from the sponsor (XXXXXXXXXX), from regulatory authorities [and from the NHS Trust(s)], where it is relevant to me taking part in this research. I give permission for these individuals to have access to my records.	
4. I consent to the central study team holding a copy of my consent form and also my contact details so that they can contact me about the study. I understand these details will be held securely and destroyed after 5 years from the end of the study.	
5. I am aware that treatment sessions may be observed for quality assurance purposes.	
6. I agree to my General Practitioner (GP) being informed of my participation in the study.	
7. I agree to be contacted for the purposes of follow up by the central GRRAND-F team who are based in XXXXXXX.	
8. I agree to take part in the GRRAND-F study.	
**OPTIONAL**	
9. I agree to take part in the optional GRRAND-F study participant interviews.	
10. I give permission that anonymous quotes from my interview may be used in the reporting of this study.	
11. I give permission for the interview to be digitally-recorded.	
12. I agree to be contacted about ethically approved research studies for which I may be suitable. I understand that agreeing to be contacted does not oblige me to participate in any further studies.	

**Name of Participant**	**Date**	**Signature**
**Name of Person Taking Consent**	**Date**	**Signature**

SupplementaryFile1.docx IRAS ID: XXXXXXX - REC reference: XXXXXX

Original consent to be filed in site file, a copy in patient notes, a copy to participant and an electronic copy for the central study office. CI: XXXXXX

Gallyer V, *et al. BMJ Open* 2021; 11:e045741. doi: 10.1136/bmjopen-2020-045741
