# Supplementary material for: Getting Recovery Right After Neck Dissection (GRRAND-F): mixed-methods feasibility study to design a pragmatic randomised controlled trial protocol
Source: BMJ Open. 2021 Jun 21;11(6):e045741. doi: 10.1136/bmjopen-2020-045741 (PMC8217923; doi:10.1136/bmjopen-2020-045741)
Supplement: Supplementary data [file bmjopen-2020-045741supp002.pdf]

Sponsor Logo

GRRAND-F
Getting Recovery Right After Neck Dissection

CONSENT FORM (GRRAND-F STUDY) PHYSIOTHERAPIST INTERVIEW STUDY

LOCAL TRUST LOGO

Name of Local Principal Investigator: ___________________________________

ID Number: G D Q - _ _ _

If you agree, please initial

1. I confirm that I have read and understood the Information Leaflet dated 10 June 2020 Version 3.0. I have had the opportunity to consider the information, ask questions and have had these answered satisfactorily.	
2. I understand that my participation is voluntary and that I am free to withdraw at any time without giving any reason, and without my legal rights being affected, any data given to the point of withdrawal would be retained.	
3. I understand that data collected during the study may be looked at by individuals from the sponsor (XXXXXXXXXXX), and from regulatory authorities, where it is relevant to me taking part in this research. I give permission for these individuals to have access to my data. I give permission for authorised individuals to have access to my data where it is relevant to this research, for a period of 5 years.	
4. I consent to the research team holding my contact details so that they can contact me about the study. I understand these details will be held securely and destroyed at the end of the study.	
5. I understand that a copy of the consent form will be kept by the local research team and a copy be sent to the central study in XXXXXXX.	
6. I give permission for anonymised written quotations from the interview to be used in reports, publications and presentations related to the study.	
7. I give permission for the interview to be digitally audio recorded.	
8. I agree to take part in this study.	

**Name of Participant**	**Date**	**Signature**
**Name of Person Taking Consent**	**Date**	**Signature**

SupplementaryFile2.docx
IRAS ID: XXXXXX - REC reference: XXXXXX CI: XXXXXXXX
Original consent to be filed in site file, a copy for physiotherapist and an electronic copy for the central study office.

Gallyer V, *et al. BMJ Open* 2021; 11:e045741. doi: 10.1136/bmjopen-2020-045741
