# Supplementary material for: Getting Recovery Right After Neck Dissection (GRRAND-F): mixed-methods feasibility study to design a pragmatic randomised controlled trial protocol
Source: BMJ Open. 2021 Jun 21;11(6):e045741. doi: 10.1136/bmjopen-2020-045741 (PMC8217923; doi:10.1136/bmjopen-2020-045741)
Supplement: Supplementary data [file bmjopen-2020-045741supp003.pdf]

Supplementary File 3: Qualitative Interview guide

Contents

- GRRAND-F patient
- GRRAND-F physio
- Care as usual patient
- Care as usual physio

GRRAND-F patient

Introduction and rapport build before beginning recording. No right or wrong answers, take you time we want to learn as much as we can from you. You are the experts. Feel free to change your mind as we go along sometimes being asked different questions can make us realise we think different things. Please ask me questions before we begin or as we are chatting, this is not a formal interview it is just us talking to understand your experience. I am an independent person and my only aim to find out what is the best way we can help people rehabilitation after NC.

1. **Do you remember at what point you were approached about being part of this study?**
    a. PROBE: cancer context (diagnosis), post-operative context and now continuing with the rest of their lives context (mortality, fear, job strain etc)
    b. How were you feeling?

2. **Can you tell me what you first thought about participating in a study like this?**
    a. PROBE: positive (benefits) or negative (concerns i.e. volume of contact query)
    b. Can you recall anything that put you off agreeing to be part of the study?
    c. And / or was there anything, in particular, which made you keen to participate?

3. **When you were approached about the study, were told that you might receive one type of programme or you might receive a different type? Can you tell me about these options?**
    a. What can you remember?
    b. What did you think/feel about these options?

4. **When you were discharged from hospital, were you given a booklet of physiotherapy exercises to take home with you? Here is a copy - Show example.**
    a. Can you remember the booklet?
    b. Did this help you to perform your physiotherapy at home?
    c. Useful?
    d. Used?
    e. How could it be improved?

5. **What did you think about the physiotherapy care you received whilst you were in hospital?**

6. **You have received X (e.g. 3) sessions of physiotherapy since your operation in X (e.g. September), can you tell me what these sessions were like?**
    a. PROBE: Can you remember any specific elements which stand out to you?
    b. Parts which were very useful for you?
    c. Made a big difference in your recovery from the surgery?
    d. How and Why?
    e. Any areas which were confusing or difficult?

7. **Can you tell me, were your appointments delivered via videocalls, or face to face or a mixture of both?**
    a. What was it like for you?
    b. Can you report any problems or difficulties you had with receiving your treatment face to face or via videocall?
        i. Probe physical
            1. e.g. did you have any technical problems with the video calls?
            2. e.g. Was it ok performing the physical movements and receiving the feedback from your physio via the video calls?
        ii. Probe psychological
            1. E.g. isolation or not feeling real at home
            2. E.g. exposing and stressful at clinic
        iii. Probe social
            1. E.g. can you have time in your home to do this or does family/others breech this privacy?

8. **Were there any sessions which you were unable to attend? Can you remember why you were unable to attend? Is there anything which the physiotherapy team could have done to make it easier for you to attend?**
    a. Can you tell me about why you were not able to attend some sessions?
        i. Physical: radiotherapy/chemotherapy side-effects, pain, function, access, time?
            1. E.g. Were you feeling too tired or in pain?
        ii. Psychological: feeling low, unmotivated
            1. E.g. did they not feel that the programme was helping them?
        iii. Social: Had to look after children/work etc, radiotherapy/chemotherapy appointments?
            1. Was it the logistics?
    b. Do you think if you had received your physiotherapy sessions face to face or via videocall that this would have helped you more?
    c. Do you think anything could be changed to help with this problem?
    d. Would you have wanted more sessions?

9. **Did you think the physiotherapy sessions have helped you recover after your operation?**
    a. We aim for the rehabilitation programme to help you to do the things you want to do to and lead the life you want.
    b. Probe physical (performing exercises, movement, fatigue, functioning?)

c. psychological (value or exercise, embarrassment of visual disfigurement, confidence)
d. social (isolated)
    i. Why do you think it helped? What has changed? Do you think it will last? What do you think you would feel like if you had not have attended these groups?
    ii. Why do you think it did not help? What would you suggest you should have been offered?

10. Can you identify any specific parts of the sessions which stood out for you? Parts which really helped? Parts you struggled with? And parts you did not understand why you were doing them?
a. Probe range of movement exercises (face neck and shoulder)- were these used?
    i. Probe how these helped
    ii. Swallowing
    iii. Upper limb mobility
b. Probe progressive resistance training were these used?
    i. Probe how these helped
    ii. Gradually increasing difficulty
    iii. Strength
c. Probe psychoeducation and behaviour change techniques aka what you talked about and some coping strategies which were used?
    i. Probe how these helped
    ii. Education e.g. positioning limbs, sleep, oral health, pain management, scar management,
    iii. exercise adherence - graded activities, fear avoidance, fatigue management, pacing, behaviour modification
    iv. promoting of independence and confidence

11. Did the physio give you an exercise diary and/ or a printed set of physiotherapy for you to complete at home? (show examples)
a. Can you remember what you received?
b. Was this helpful? Can you describe how you used it (if you did)?
c. Why and why not
    i. Probe capability:
        1. Physical: physically able to perform them?
        2. Psychological: did you feel that you were able to perform them?
    ii. Opportunity:
        1. Physical: Did you have space, time to perform physio exercises at home. Did you use the diary was it helpful?
        2. Social: family/friends support or not help i.e. not giving you space/time?
    iii. Motivation:
        1. Reflective: Did you think it was worth it?
        2. Automatic: worries about performing exercises?

12. **You completed a set of questionnaires before and after completing the GRRAND-F programme. What did you think about these questions? (*Share the questionnaires to remind if nothing is remembered*).**
    a. Do they capture the issues which you think are important to you or were any issues that you think have been missed?
        i. Probe physical, psychological and social issues
    b. Were there any which you found difficult to complete?
    c. Any which you did not like?
    d. Were there too many or too few questionnaires?
    e. Did you complete them all and if not can you explain why – could the research team change them to make them better?
    f. Would you have liked to have used physical measures to test if your strength had improved?

13. **Have you sought any other type of help during your rehabilitation? outside of what we have offered you in this trial?**
    a. Paid for other therapists?
    b. Been referred within the NHS?

14. **Do you have any other feedback you would like to talk about.**
    a. Things which we could change in how we deliver the programme?
    b. What is in the programme?
    c. How many sessions you receive?
    d. What happens once you have finished the programme?

GRRAND-F Physio

Introduction and rapport build before beginning recording. No right or wrong answers, take you time we want to learn as much as we can from you. You are the experts. Feel free to change your mind as we go along sometimes being asked different questions can make us realise we think different things. Please ask me questions before we begin or as we are chatting, this is not a formal interview it is just us talking to understand your experience. I am an independent person and our only aim to find out what is the best way we can help people rehabilitation after NC.

1. **What has it been like being part of this research study?** (Opening broad question see what is the most pertinent issues which arise)
    a. Probe differences between different sites
    b. Difficulties and benefits
    c. Things you had wished you had known before agreeing to be part of the trial?

2. **Have you worked with this patient group (i.e. HNC NC rehab) before?**
    a. Can you tell me how you felt before the study began? Any concerns?
    b. How you feel now you have been working with this group
    c. If you have been working with this groups previously, can you tell me if the patients who agreed to be part of this study were similar or different to the patients you have seen before?

3. **Can you tell me about the training you received before participating in this study?**
    a. Bests bits
    b. Bits to change
    c. Bits to add
    d. Needed more / less?

4. **After you received your training in the GRRAND-F intervention, did you think this programme would help patients?**
    a. Can you explain to me why/not?
    b. If you could change this programme what would you include/remove?
        i. Probe physical, psychological and social needs of patients

5. **Did you deliver the physiotherapy via videocalls, or face to face or a mixture of both?**
    a. What was it like for you?
    b. Barriers/problems and facilitators with either modality
        i. Probe physical (observing exercises, technical issues)
        ii. Probe psychological (connection?)
        iii. Probe social
    c. Did you have appropriate space to deliver the GRRAND-F groups either via videocalls or face to face at your place of work

6. **Did you give you patients exercise diaries to monitor the physiotherapy they did at home?**

a. Did you think these were useful for you to know what was going on?
b. Did you think they helped your patients?
c. Can you offer any suggestions of how to change them?

7. **Did you give your patients handouts of physiotherapy activities for them to use at home?**
    a. Were these useful?
    b. Do you think they were they used?
    c. Can you suggest any improvements?

8. **Do you think/know that your patients practiced their physiotherapy exercises between sessions? Is there anything which you can suggest that the team do to improve adherence?**
    a. Why and why not
        i. Probe capability: physical and psychological
            1. Do the patients understand and appreciate how important to their recovery it is to perform these physio exercises?
            2. Do they believe that they can perform these physio exercises?
        ii. Opportunity: probe physical and social
            1. Handouts to show them how to perform physio exercises
            2. Do they have time and support to do these rehab exercises?
        iii. Motivation: probe reflective and automatic
            1. Patients believe
            2. Patients fearful

9. **Did you experience many DNA and UTA appointments?**
    a. Were these videocall or face to face appointments?
    b. Do you remember why your patients were unable to attend?
    c. Why do you think that was?
    d. Could we do anything to change the trial or intervention to alleviate this problem?

10. **If you focus on the contents of the GRRAND-F intervention now, what do you think are the most useful elements and any suggestions for changes? Can you talk me through what you think of…**
    a. The range of movement exercises (face neck and shoulder)- were these used often, most??
        i. Probe how these helped
        ii. Swallowing
        iii. Upper limb mobility
    b. Probe progressive resistance training were these used?
        i. Probe how these helped
        ii. Gradually increasing difficulty
        iii. Strength
    c. Probe psychoeducation and behaviour change techniques aka what you talked about and some coping strategies which were used?
        i. Probe how these helped

ii. Education e.g. positioning limbs, sleep, oral health, pain management, scar management,
iii. exercise adherence - graded activities, fear avoidance, fatigue management, pacing, behaviour modification
iv. promoting of independence and confidence

11. What do you think are the major barriers to implementing an intervention such as this into usual care?

a. Workload
b. Negative consequences?
c. Could we adapt it to suit your local service needs more?

12. Do you think this programme has helped your patients?

a. We aim for the rehabilitation programme to help your patients do the things they want to do to and lead the life they want.
    i. Probe physical (performing exercises, movement, fatigue, functioning?)
    ii. psychological (value or exercise, embarrassment of visual disfigurement, confidence)
    iii. social (isolated)
b. Why do you think it helped? What has changed? Do you think it will last? What do you think they would feel like if they had not have attended these groups?
c. Why do you think it did not help? What would you suggest you should have been offered?
    i. Probe for specific ideas

13. **Do you have any other feedback you would like to talk about.**

a. Things which we could change in how we deliver the programme?
b. What is in the programme?
c. How many sessions patients receive?
d. What happens once your patients have finished the programme?
e. Or any other comments?

Care as usual patient

Introduction and rapport build before beginning recording. No right or wrong answers, take you time we want to learn as much as we can from you. You are the experts. Feel free to change your mind as we go along sometimes being asked different questions can make us realise we think different things. Please ask me questions before we begin or as we are chatting, this is not a formal interview it is just us talking to understand your experience. I am an independent person and our only aim to find out what is the best way we can help people rehabilitation after NC.

1. **Do you remember at what point you were approached about being part of this study?**
    a. PROBE: cancer context (diagnosis), post-operative context and now continuing with the rest of their lives context (mortality, fear, job strain etc)
    b. How were you feeling?

2. **Can you tell me what you first thought about participating in a study like this?**
    a. PROBE: positive (benefits) or negative (concerns i.e. volume of contact query)
    b. Can you recall anything that put you off agreeing to be part of the study?
    c. And / or was there anything, in particular, which made you keen to participate?

3. **When you were approached about the study were told that you might receive one type of physiotherapy or you might receive a different type. Can you tell me about these options?**
    a. What can you remember?
    b. What did you think/feel about these options?

4. **Can you tell me about the physiotherapy you received during this trial?**
    a. Was this what you were expecting?
    b. Did you hope to be in one group or another?
    c. How did you feel once you learnt what type of rehabilitation you would be receiving?

5. **When you were in hospital after your operation, do you remember the advice you received from the physiotherapist who worked with you?**
    a. What do you remember from the advice?
    b. What did you think about the advice?
    c. What would you like to change? Or stay the same?

6. **When you were discharged from hospital after your operation, did you receive a booklet of physiotherapy exercises and an exercise diary to take home with you?**
    a. Can you tell me what you thought about these?
    b. Were they useful?
    c. Have you performed any of these exercises?
    d. Do you think these should always be given out or not?

d. Did you complete them all and if not can you explain why – could we change them?

7. **Did you think the advice you received in hospital and the booklet you took home with you helped you with your recovery?**
    a. We aim for the rehabilitation programme to help you to do the things you want to do to and lead the life you want.
    b. Probe physical (performing exercises, movement, fatigue, functioning?)
    c. psychological (value or exercise, embarrassment of visual disfigurement, confidence)
    d. social (isolated)
        i. Why do you think it helped? What has changed? Do you think it will last?
        ii. Why do you think it did not help? What would you suggest you should have been offered?

8. **Did you perform the physiotherapy and follow the advice in the booklet**? Did you use the exercise diary?
    a. Why and why not
        iii. Probe capability: physical and psychological
        iv. Opportunity: probe physical and social
        v. Motivation: probe reflective and automatic

9. **Have you sought any other therapy outside of what this trial provided to help you in your rehabilitation?**
    a. Referral within NHS
    b. Use of private services outside of NHS

10. **You completed a set of questionnaires (*Share the questionnaires to remind if nothing is remembered*). What did you think about these questions?**
    a. Do they capture the issues which you think are important to you or were any issues that you think have been missed?
        vi. Probe physical, psychological and social issues
    b. Were there too many or too few questionnaires?
    c. Were there any you did not like? Did not wish to complete?
    d. Would you expect or want am objective measurement of physical strength to see if it is changing?

11. **Do you have any other feedback you would like to talk about.**
    a. Did you seek any other advice/help outside of the programme? Or did you feel like you needed to?
    b. Things which we could change in how we deliver the programme?
    c. What is in the programme?
    d. How many sessions you receive?
    e. What happens once you have finished the programme?
    f. Or any other comments?

Care as usual physio

Introduction and rapport build before beginning recording. No right or wrong answers, take you time we want to learn as much as we can from you. You are the experts. Feel free to change your mind as we go along sometimes being asked different questions can make us realise we think different things. Please ask me questions before we begin or as we are chatting, this is not a formal interview it is just us talking to understand your experience. I am an independent person and our only aim to find out what is the best way we can help people rehabilitation after NC.

1. **What has it been like being part of this research study?** (Opening broad question see what is the most pertinent issues which arise)
    a. Probe differences between different sites
    b. Difficulties and benefits
    c. Things you had wished you had known before agreeing to be part of the trial?

2. **Have you worked with this patient group (i.e. HNC NC rehab) before?**
    a. Can you tell me how you felt before the study began? Any concerns?
    b. How you feel now you have been working with this group
    c. If you have been working with this groups previously, can you tell me if the patients who agreed to be part of this study were similar or different to the patients you have seen before?

3. **Can you tell me about the training you received before participating in this study?**
    a. Bests bits
    b. Bits to change
    c. Bits to add
    d. Needed more / less?

4. **After you received your training, did you think the advice and information you were going to give to your patients would help them a lot, a little or not much?**
    a. Can you explain to me why/not?

5. **Is the advice and information you delivered to the patients very different from what you usually do with this patient group?**

6. **Did you give your patients the booklet and exercise diaries so that they could monitor their exercises at home?**
    a. Did you think the discharge booklet was useful?
    b. Did you think the exercise diary was useful?
    c. Did you think they helped your patients?
    d. Can you offer any suggestions of how to change them?

7. **Do you think the advice and information has helped your patients a lot, a little or not much?**
    a. Can you explain why or why not?

8. **Do you have any other feedback you would like to talk about.**
    a. Things which we could change in how we run the study?
    b. What happens once your patients have finished the programme?
    c. Or any other comments?
