# Supplementary material for: Getting Recovery Right After Neck Dissection (GRRAND-F): mixed-methods feasibility study to design a pragmatic randomised controlled trial protocol
Source: BMJ Open. 2021 Jun 21;11(6):e045741. doi: 10.1136/bmjopen-2020-045741 (PMC8217923; doi:10.1136/bmjopen-2020-045741)
Supplement: Author's manuscript [file bmjopen-2020-045741.draft_revisions.pdf]

BMJ Open

BMJ Open is committed to open peer review. As part of this commitment we make the peer review history of every article we publish publicly available.

When an article is published we post the peer reviewers' comments and the authors' responses online. We also post the versions of the paper that were used during peer review. These are the versions that the peer review comments apply to.

The versions of the paper that follow are the versions that were submitted during the peer review process. They are not the versions of record or the final published versions. They should not be cited or distributed as the published version of this manuscript.

BMJ Open is an open access journal and the full, final, typeset and author-corrected version of record of the manuscript is available on our site with no access controls, subscription charges or pay-per-view fees (http://bmjopen.bmj.com).

If you have any questions on BMJ Open's open peer review process please email info.bmjopen@bmj.com

BMJ Open

Getting Recovery Right After Neck Dissection (GRRAND-F): mixed-methods Feasibility study to design a pragmatic randomised controlled trial Protocol

Journal:	*BMJ Open*
Manuscript ID	bmjopen-2020-045741
Article Type:	Protocol
Date Submitted by the Author:	13-Oct-2020
Complete List of Authors:	Gallyer, Victoria; University of Oxford, Nuffield Department of Orthopaedics, Rheumatology and Musculoskeletal Sciences (NDORMS)<br>Smith, Toby O.; University of Oxford , Nuffield Department of Orthopaedics, Rheumatology and Musculoskeletal Sciences; University of East Anglia,<br>Fordham, Beth; University of Oxford , Nuffield Department of Orthopaedics, Rheumatology and Musculoskeletal Sciences (NDORMS)<br>Dutton, Susan; University of Oxford, Centre for Statistics in Medicine, Nuffield Department of Orthopaedics, Rheumatology and Musculoskeletal Sciences (NDORMS)<br>Chester-Jones, Mae; University of Oxford, Centre for Statistics in Medicine, Nuffield Department of Orthopaedics, Rheumatology and Musculoskeletal Sciences (NDORMS)<br>Lamb, Sarah; University of Exeter, College of Medicine and Health; University of Oxford, NDORMS<br>Winter, Stuart; University of Oxford, Nuffield Department of Surgical Sciences
Keywords:	Head & neck tumours < ONCOLOGY, Head & neck surgery < SURGERY, REHABILITATION MEDICINE

SCHOLARONE™
Manuscripts

I, the Submitting Author has the right to grant and does grant on behalf of all authors of the Work (as defined in the below author licence), an exclusive licence and/or a non-exclusive licence for contributions from authors who are: i) UK Crown employees; ii) where BMJ has agreed a CC-BY licence shall apply, and/or iii) in accordance with the terms applicable for US Federal Government officers or employees acting as part of their official duties; on a worldwide, perpetual, irrevocable, royalty-free basis to BMJ Publishing Group Ltd ("BMJ") its licensees and where the relevant Journal is co-owned by BMJ to the co-owners of the Journal, to publish the Work in this journal and any other BMJ products and to exploit all rights, as set out in our licence.

The Submitting Author accepts and understands that any supply made under these terms is made by BMJ to the Submitting Author unless you are acting as an employee on behalf of your employer or a postgraduate student of an affiliated institution which is paying any applicable article publishing charge ("APC") for Open Access articles. Where the Submitting Author wishes to make the Work available on an Open Access basis (and intends to pay the relevant APC), the terms of reuse of such Open Access shall be governed by a Creative Commons licence – details of these licences and which Creative Commons licence will apply to this Work are set out in our licence referred to above.

Other than as permitted in any relevant BMJ Author's Self Archiving Policies, I confirm this Work has not been accepted for publication elsewhere, is not being considered for publication elsewhere and does not duplicate material already published. I confirm all authors consent to publication of this Work and authorise the granting of this licence.

For peer review only

Getting Recovery Right After Neck Dissection (GRRAND-F): mixed-methods Feasibility study to design a pragmatic randomised controlled trial Protocol

Authors: Victoria H Gallyer MSc[1], Toby O Smith PhD[1,2], Bethany A Fordham PhD[1], Susan J Dutton MSc[3], Mae Chester-Jones MSc[3], Sarah E Lamb DPhil[1,4], Stuart C Winter MD, FRCS[5] on behalf of the GRRAND-F Trial Collaborators.

Affiliations

[1] Centre for Rehabilitation Research in Oxford, Nuffield Department of Orthopaedics, Rheumatology and Musculoskeletal Sciences (NDORMS), University of Oxford, UK

[2] Faculty of Medicine and Health Sciences, University of East Anglia, Norwich, UK

[3] Centre for Statistics in Medicine, Nuffield Department of Orthopaedics, Rheumatology and Musculoskeletal Sciences (NDORMS), University of Oxford, UK

[4] College of Medicine and Health, University of Exeter, UK

[5] Nuffield Department of Surgical Sciences, University of Oxford. Oxford University Hospitals NHS Foundation Trust, UK

Corresponding Author: Mr Stuart Winter, Department of Head and Neck Surgery, Oxford University Hospitals NHS Foundation Trust, Churchill Hospital, Headington, Oxford, UK stuart.winter@nds.ox.ac.uk. Telephone: 01865 227138, Fax: 01865 231091

Keywords: Head and neck cancer, Neck dissection, Rehabilitation, Physiotherapy

Word Count: 4333

For peer review only

For peer review only - http://bmjopen.bmj.com/site/about/guidelines.xhtml

ABSTRACT

INTRODUCTION: We will evaluate the feasibility of a randomised controlled trial (RCT) to estimate the effectiveness and cost-effectiveness of a rehabilitation intervention on pain, function and health-related quality of life following neck dissection (ND) after head and neck cancer (HNC).

METHODS AND ANALYSIS: This is a pragmatic, multicentred, feasibility study. Participants are randomised to usual care (control) or usual care plus an individualised, rehabilitation programme (GRRAND Intervention). Adults aged over 18 with HNC for whom neck dissection is part of their care will be recruited from specialist clinics. Participants are randomised in 1:1 ratio using a web-based service. The target sample size is 60 participants. Usual care will be received by all participants during their post-operative inpatient stay consisting standard NHS care supplemented with a booklet advising on post operative self-management strategies. The GRRAND intervention programme consists of usual care plus up to six individual physiotherapy sessions including range of motion, progressive resistance exercises, advice, education and discussion. Between sessions participants will be advised to complete a home exercise programme. The primary outcome is to determine recruitment and retention rates from study participants across sites. Outcomes will be measured at six and 12 months . Participants and physiotherapists will be invited to an optional qualitative interview at the completion of their involvement in the study. The target qualitative sample size is 15 participants and 12 physiotherapists. Interviews aim to further investigate the feasibility and acceptability of the intervention and to determine wider experiences of the study design and intervention from patient and physiotherapist perspectives.

For peer review only - http://bmjopen.bmj.com/site/about/guidelines.xhtml

ETHICS AND DISSEMINATION: Ethical approval was given on 29 October 2019 (National Research Ethics Committee Number: 19/SC/0457). Results will be reported at conferences and in peer-reviewed publications.

TRIAL ISRCTN REGISTRATION NUMBER: 11979997

STATUS: trial recruitment is ongoing and is expected to be completed by 30th Aug 2021.

Strengths and limitations of this study:

- GRRAND-F (Getting Recovery Right After Neck Dissection) is a pragmatic, multicentred, randomised control feasibility trial.
- We will evaluate whether it is feasible to run a RCT to assess the effectiveness and cost-effectiveness of a rehabilitation intervention in improving pain, function and health-related quality of life following ND after HNC.
- The primary outcome is recruitment and retention rates.
- The qualitative sub-study will explore the wider experiences and perceptions of the study design and intervention from a patient and physiotherapist perspective.

For peer review only

For peer review only - http://bmjopen.bmj.com/site/about/guidelines.xhtml

INTRODUCTION

Head and neck cancer (HNC) affects 700,000 people worldwide and over 11,000 in the UK annually[1-3]. HNC refers to neoplasms at different anatomical sites. Within the UK, tumours of the oropharynx are the most common and have seen a two-fold increase in incidence over the last 20 years, largely attributed to human papillomavirus (HPV)[4,5]. During this time there has also been a 30% increase in oral cancer[4-6]. While there has been a significant increase in HNC, prognosis and survival in the UK continues to improve[4,6]. Therefore the proportion of people living with the effects of this cancer and its treatment continues to increase.

The treatment pathway for HNC is complex, due to the varied anatomical sites of disease and the needs of the patient. Treatment for HNC requires treatment of the primary site, as well as the neck when there is spread to the lymph nodes or high probability of spread. Historically almost all patients received a neck dissection (ND). With the advent of chemo-radiotherapy as a curative treatment, less patients require a ND. However even with this approach, up to 20% of patients require a ND due to residual disease[6]. Side-effects from surgery can be significant, including swallowing problems, neck and shoulder problems, difficulties sleeping, fatigue and anxiety[7,8].

Post-operative complications are common following ND[8-11]. Early complications can include shoulder pain and infection. Late complications may not appear until 3 months post-treatment, and can continue to present over five years[12,13]. These complications include shoulder movement dysfunction, speech, swallowing and musculoskeletal problems such as cervical contracture and muscle wastage[12]. Psychosocial complications are also highly

For peer review only - http://bmjopen.bmj.com/site/about/guidelines.xhtml

prevalent post-operatively, predominantly fatigue, anxiety, depression, sleep disturbance and social isolation. Sequelae of shoulder dysfunction and psychosocial complications are strongly associated with reduced return to work, with up to 50% of patients ceasing working due to shoulder disability alone[10,14].

Rehabilitation was one of 22 key questions in the 2016 National Institute for Health and Care Excellence (NICE) Clinical Guideline[15] on the management of HNC. The guideline recommends clinicians “consider progressive resistance training for people with impaired shoulder function, as soon as possible after ND”. The review noted that this evidence was from small trials with a high risk of bias. The review also highlighted a knowledge gap on how to rehabilitate HNC patients’ wider side-effects. The NICE guideline concluded that a prospective randomised trial was required to understand how best to promote recovery following HNC, making this a recognised National Health Service (NHS) research priority[15].

Currently there is no national standard best practice for rehabilitation following HNC. Our study development work[16] and feedback from patient and public (PPI) representatives has shown that physiotherapy practice varies across the UK. The findings suggested that rehabilitation in the form of physiotherapy is not routinely available to patients with HNC, in either in-patient or outpatient settings[16]. When rehabilitation is offered it is often not evidence-based, and targets acute respiratory care, range of motion (ROM) exercises for the neck and shoulder, and advice on positioning of the upper limb and shoulder girdle[15]. A booklet may be provided to supplement this treatment. Outpatient treatment is minimal, and most commonly reactive, driven by patient request. Therefore the current evidence-base is limited in quality and only focuses on shoulder rehabilitation. There remains a gap in

For peer review only - http://bmjopen.bmj.com/site/about/guidelines.xhtml

knowledge on how to rehabilitate patient's wider side-effects following surgery for HNC such as fatigue, anxiety, poor sleep and return to work. Consequently, both Cochrane[17] and NICE[15] concluded that further high-quality research is needed to determine how best to promote recovery for shoulder function, quality of life and cost-effectiveness of treatment.

This study will evaluate whether it is feasible to run a RCT to assess the effectiveness and cost-effectiveness of a rehabilitation intervention in improving pain, function and health-related quality of life following ND after HNC. In addition to investigating the feasibility of an enhanced rehabilitation intervention following HNC ND, this trial will also standardise usual care.

METHODS AND ANALYSIS

Trial Design

A mixed-methods feasibility study investigating the design of a RCT to test the clinical and cost-effectiveness of usual care and an individualised, rehabilitation programme compared to usual care alone in patients undergoing a ND for HNC. The study flow chart is presented as **Figure 1**. **Table 1** presents a summary of trial objectives, outcome measures and time points.

Eligibility

Participants are eligible to take part in the trial if they fulfil the eligibility criteria listed in **Box 1**.

Recruitment

For peer review only - http://bmjopen.bmj.com/site/about/guidelines.xhtml

Potential participants will be identified from UK NHS hospital trusts as requiring a ND as part of their treatment, and will be approached by a member of the clinical team to ask whether they would like to know more about the GRRAND-F study.

They will be asked to read the Patient information sheet (PIS) and to discuss their potential participation with anyone who they feel would provide useful advice. Potential participants will also be provided with contact information for the research team who will be able to answer any questions relating to the study. The number of patients provided with the PIS will be recorded to monitor the number of patients who are approached.

Eligible patients who agree to participate will then be asked to provide their written informed consent (**Supplementary File 1**).

Randomisation, Blinding and Allocation Concealment

Following the completion of the consent process baseline data will be collected. Participants will then be randomised once their eligibility has been confirmed post-operatively prior to hospital discharge.

Participants will be randomised in a 1:1 ratio using the centralised web-based randomisation service provided by Oxford Clinical Trials Research Unit (OCTRU). Randomisation will be undertaken using minimisation to ensure balanced allocation of participants across the two treatment groups, stratified by hospital site and spinal accessory nerve sacrifice.

The minimisation algorithm will incorporate a non-deterministic element and will be seeded using simple randomisation to prevent predictability in the early stages of the study.

Due to the nature of the intervention, participants and clinicians delivering physiotherapy will not be blinded to treatment allocation.

For peer review only - http://bmjopen.bmj.com/site/about/guidelines.xhtml

Intervention

Usual Care

Usual care will be received by both control and experimental intervention groups.

As part of usual care, all participants will receive the same in-patient rehabilitation programme, commencing day one post-operatively (or next physiotherapy working day), consisting of:

(1) Advice to practise simple ROM exercises for the face and neck for the purpose of preventing the onset of post-surgical contracture and optimising swallowing and shoulder movement.

(2) Respiratory care, targeting sputum clearance and breathing control.

(3) Education on body positioning to reduce pressure and pull on the shoulder girdle, oral health to reduce food pocketing in the mouth, and pain management and pacing activities to optimise levels of comfort and function.

The content, dosage and timing of in-patient physiotherapy contact will be recorded.

Reflecting usual care, on discharge participants will receive a booklet providing advice on post-operative self-management strategies including exercise, pain management, return to work and activities of daily living. This has been developed by the multidisciplinary trial team and collaborations with two of the participating NHS centres in Birmingham and Oxford to ensure that the information is standardised. Reflecting current practice, once discharged from hospital, physiotherapy will not be routinely provided to these participants.

For peer review only - http://bmjopen.bmj.com/site/about/guidelines.xhtml

Experimental intervention

Participants randomised to this group will receive the same in-patient rehabilitation programme as participants in the Usual Care Group *PLUS* an individualised rehabilitation programme. This will be delivered by a GRRAND-F-trained physiotherapist in an outpatient setting. In the event that the participant is still an in-patient, this will be commenced in hospital and continued, post-discharge, in an outpatient setting. The frequency to which this change of setting occurs will be recorded as part of the feasibility outcomes.

At the initial consultation, physiotherapists will assess the participant to identify modifiable physical and psychosocial factors associated with poor recovery following HNC surgery. These may include: muscle weakness, limited ROM, reduced sensation, pain and fear avoidance beliefs. Based on this assessment, physiotherapists will prescribe from a pre-specified range of rehabilitation options (see **Figure 2**).

Programmes will be individualised to contain one, several, or all of the treatment options, dependent on participant's needs. Participants will also be provided with a home exercise programme to supplement face-to-face sessions.

Individualised Rehabilitation Options

(1) ROM exercises targeting muscles and joints of the face, neck and shoulder impacted by ND. The purpose of these exercises is the prevention of post-surgical contracture, and the maintenance of swallowing and upper limb mobility.

(2) Progressive resistance exercises, targeting strengthening of the neck and shoulder. Resistance loads will initially be set at a moderate level of exertion (based on the modified Borg scale of perceived exertion [18]) to permit progression, enhance motivation and

For peer review only - http://bmjopen.bmj.com/site/about/guidelines.xhtml

adherence, and reduce the possibility of symptom flare-up. Resistance will consist resistance bands at the shoulder and isometric resistance provided by the participant’s hand for neck and temporomandibular joint exercises.

Exercises will be progressed by increasing the resistance load, speed, number of repetitions and sets or by progressing the range in which the exercise is completed and through the introduction of weight-bearing exercises through the upper limb. Additionally, the exercises will become increasingly ‘task specific’, targeting participant’s specific functional goals.

(3) Education and advice on a number of recognised potential post-operative complications including:

- Positioning limbs to prevent joint contractures
- Oral health particularly for patients following upper cervical/head/oral surgery
- Pain management for both early and later post-operative stages through positioning, taking prescribed analgesics and pacing/behaviour modfication.
- Scar management.
- Exercise adherence and return to function with fatigue management and pacing of activities
- Promote independence and confidence to return to normal activities of daily living, work, and social pursuits.

This will be delivered through the introduction of techniques of goal setting, fear avoidance, pacing and fatigue management, behaviour modification and graded activity. This has been successfully taught and delivered by the research team in previous NIHR trials (BOOST[19], DAPA[20]), to provide a basis for this new intervention. Advice will be provided through

For peer review only

For peer review only - http://bmjopen.bmj.com/site/about/guidelines.xhtml

discussion during consultations and re-enforced with worksheets designed by the multi-disciplinary trial team.

The intervention may be modified in the development phase of the trial. The intervention will be finalised prior to the main trial. If there are no substantive changes, participants will contribute to the main trial analysis.

Delivery

The experimental intervention will be delivered a maximum of six sessions over a six-month period. The design will enable assessment of how many sessions are required.The first session will aim to occur within 14 days of surgery. Reflecting normal NHS practice, the initial session will be 60 minutes, and subsequent sessions up to 45 minutes in duration. The physiotherapist, in collaboration with the participant, will agree the spacing of sessions, reflecting normal clinical practice. This spacing will allow for maximum progression of the intensity of exercise over a time period sufficient to (hypothetically) produce an improvement in outcome. Treatment options may also be added or removed at each session, in line with the participant’s current treatment progress and health status.

Contamination

The GRRAND-F physiotherapists who deliver the experimental intervention sessions where possible will not deliver physiotherapy to those in the control group (and vice versa). The details of the physiotherapists delivering sessions will be recorded and reviewed to monitor this risk of contamination. Due to the interventions being individualised and delivered in an

For peer review only

For peer review only - http://bmjopen.bmj.com/site/about/guidelines.xhtml

outpatient setting, there is a low risk of participants sharing their knowledge and experience between groups, further minimising the risk of between-group contamination.

Co-Interventions

Respecting the pragmatic nature of this study design, participants from either group will not be asked to desist from receiving any other forms of treatment during the trial or follow-up periods. If a participantreceives additional treatment, the details of the treatment received and the reasons for administering will be collected.

Quality Assessment

The trial will be monitored and audited in accordance with the current approved protocol, good clinical practice[21], relevant regulations and standard operating procedures (SOPs).

All designated physiotherapists who deliver usual care will be taught the standardised control intervention procedures.

Physiotherapists delivering the GRRAND intervention will attend a face-to-face training session where they will be taught the intervention and processes involved by a member of the GRRAND-F team who developed the intervention (TS, VG). Each intervention physiotherapist will be monitored during a site visit at their third/fourth session. Sessions will be monitored against the protocol to determine whether there are issues around fidelity, contamination across groups or adherence/compliance of participants. Where further training or further monitoring visits are required, these will be instigated following these visits.

Assessments

For peer review only

Data will be clinical and participant-reported and collected using questionnaires at baseline and six months post-randomisation. Data will also be collected for those participants who reach 12-month follow-up during the data collection phase. This is estimated to be applicable for up to 50% of the cohort. Data will be collected alongside routine clinical appointments at each site. A primary end-point of six-months post-randomisation was chosen to provide a signal on clinical outcomes after completing the intervention. The 12-month data provides data to assess the risk of attrition and missing data at 12 months, which will assist with the development of the definitive trial if it proves to be feasible.

Baseline Assessment

Baseline data will be collected prior to randomisation once consent has been obtained, typically during the pre-operative assessment.. Data collection is described in **Table 2**.

Outcome data to be collected at each of the data collection intervals are listed below.

- Shoulder pain and function measured using the well-validated Shoulder Pain and Disability Index (SPADI)[22, 23].
- Pain measured using the SPADI 5-item Pain Sub-scale[23] and a Numerical Rating Scale.
- Function measured using the SPADI 8-item Function sub-scale[23]
- Pain medication details and usage relating to head, neck and shoulder.
- Chemotherapy and radiotherapy treatment provision.
- Health-related quality of life measured using the EQ-5D-5L score[24] and the EORTC questionnaires (C30 (core)[25] and H&N43 (head and neck specific)[26,27]).

For peer review only - http://bmjopen.bmj.com/site/about/guidelines.xhtml

- Health resource use questionnaire (collection of health resources for computation of direct medical, direct nonmedical and indirect costs); additional out-of-pocket expenses; and work absence.
- Physical performance measures including shoulder and neck ROM and grip strength will be measured by an appropriately trained member of the research team.
- Adverse events: such as prolonged delayed onset muscle soreness, swelling and wound irritation.

Follow-up procedures

Data will be collected from participants at six and 12-months (if applicable) from date of surgery with a target of +/- 1 month, at their routine NHS check-up appointments. If participants do not attend their follow-up appointment, they will be contacted by telephone, and, if appropriate, sent the questionnaires to complete. The study team will attempt to telephone these participants on up to two occasions. If these methods fail, we will categorise the participant as a 'non-responder' for that time-point only. The data collection schedule is presented in **Table 2**.

Outcome Measures

Feasibility outcome data to be collected will include:

- Screening log numbers of eligible patients, including reasons for exclusion/non-participation.

For peer review only - http://bmjopen.bmj.com/site/about/guidelines.xhtml

- Recruitment numbers and rate; overall and per site.
- Protocol adherence, including fidelity to control and experimental interventions using treatment logs, timing and location of intervention delivery (in particular the first session) alongside frequency of physiotherapy contact. This will assist in assessing both potential between-group contamination and intervention delivery. We will also monitor the intervention delivery as part of the Quality Assurance (QA) monitoring visits. The findings of these visits will provide data on intervention location, fidelity to the protocol, and barriers or facilitators to provision across the sites.
- Follow-up completion rate and overall study retention in each study arm for the outcome measures highlighted above.

The primary and secondary outcome measures for this trial are presented in **Table 1**.

Data Analysis

Sample Size

As this is a feasibility study which is not aimed to assess treatment effects, we have not undertaken a formal power sample size calculation.

Sixty participants will be recruited, based on Teare et al's recommendation[28] that between 50 and 70 are required when continuous scale data outcomes are to be collected. This assumes a 10% drop-out. This will also provide sufficient data to answer our feasibility objectives with 30 participants from each group recruited. Based on 2017 data from two of

For peer review only - http://bmjopen.bmj.com/site/about/guidelines.xhtml

the participating sites, approximately 160 potentially eligible participants were identified. Based on a conservative judgement of 45% recruitment rate for this rehabilitation trial with this cohort[17,29,30], over 60 participants could be recruited within a 12-month period. This is within the required number to conduct this study.

Statistical Analysis

Recruitment and follow-up rates are the main drivers for the feasibility design on the basis that unless reasonable rates can be achieved no formal trial will be possible. Recruitment rate will be calculated as the number of participants randomised as a proportion of eligible participants. Rates will be estimated based on data collected and a 95% confidence interval determined for these measures. The rate of incomplete information either due to drop-out to the interventions or non-completion of the outcome measures will be based on the number of participants randomised. The statistical analysis will also estimate, with 95% confidence intervals, the parameters required for a formal power calculation, particularly the standard deviation of potential outcome measures.

If the estimated recruitment and follow-up rates are such that a multicentre definitive trial is possible no formal analysis will be undertaken and data from the feasibility will be locked and carried over into the definitive trial, where funding for the definitive trial has been obtained. In this case no formal analysis of treatment efficacy will be undertaken. The definitive trial will be planned based on the data collected during this feasibility study. The mean difference, standard deviation and effect size with between-group inferential statistical analyses will be estimated to determine direction and magnitude of effect and to inform a power calculation for a definitive trial.

For peer review only - http://bmjopen.bmj.com/site/about/guidelines.xhtml

The 'traffic light' system will be used as a guide for progression to a definitive trial (**Table 3**)[31].If any of the criteria are not met, these will be discussed by the Trial Steering Committee (TSC) to decide if a definitive trial is feasible.

Descriptive statistics will be used to describe the demographics between the two groups. Clinical outcome data will be reported depending on the type of variable: for continuous variables the means and standard deviation in each group (or median and interquartile range if non-normally distributed) together with the unadjusted and adjusted difference in means and corresponding 95% confidence intervals with analysis of covariance, adjusting for baseline values (where appropriate) and stratification factors; for categorical variables, the number and percentage of participants in each category will be reported and unadjusted and adjusted odds ratios (for binary outcomes) together with their 95% confidence intervals will be reported.

All results will be based on the intention-to-treat population. Protocol deviations will be reported as these are an important part of the feasibility assessment when planning the definitive trial.

For peer review only

Health Economics

Data on health care utilisation will be collected but not analysed. To answer the feasibility questions related to the health economic perspectives, we will test the completion of the health resource use questionnaire and will present the data descriptively.

Data Management

For peer review only - http://bmjopen.bmj.com/site/about/guidelines.xhtml

All data will be processed according to the Data Protection Act 2018[21,32,33] and all documents will be stored safely in confidential conditions. Trial-specific documents, except for the signed consent form and contact details, will refer to the participant with a unique study participant number and initials only. Participant identifiable data will be stored separately from trial data.

Qualitative Investigation

The embedded qualitative study will assess the feasibility and acceptability of the experimental and control interventions from the perspectives of those delivering (physiotherapists) and receiving (participants) the interventions. The format and delivery of the qualitative interviews are based on parameters successfully implemented in previous trials conducted by the research team (BeST[34], BOOST[19], PROSPER[35], SARAH[36]), and UK trials involving cancer patients[37]. Specifically, participant opinion and experience of study recruitment, intervention content, timing, and accessibility and barriers and facilitators to adherence will be sought. Qualitative themes identified will be used to modify the content and delivery of a future definitive trial.

<u>*Recruitment*</u>

Fifteen participant interviews will be conducted, involving 10 participants from the experimental intervention group and five from the control group. Based on our previous trial work[34,36], this sample size is expected to ensure data saturation across both groups, allowing for the expected larger dataset from the experimental intervention group.

For peer review only

For peer review only - http://bmjopen.bmj.com/site/about/guidelines.xhtml

All participants will be given a brief explanation of the interviews during the initial consent process. Those willing to be interviewed will indicate permission to be contacted by the qualitative researcher on the Consent Form (**Supplementary File 1**). It will be clarified that not all willing participants may be required for the interview study.

Participants who have agreed to be contacted for the interview will be purposively sampled by the qualitative researcher to ensure the 15 interview participants are demographically representative of the full study sample. Targeted demographics include age, ethnicity, employment status, and extent of ND.

The qualitative researcher will telephone the sampled participants, and answer any questions they may have about taking part in the interviews. If the participant agrees to take part, a time and date convenient to the participant will be arranged for an interview. Interviews will be conducted face-to-face, and occur at a location convenient to the participant, most likely in their own home.

A minimum of one physiotherapist who delivered the experimental intervention and one physiotherapist who delivered the control intervention will be interviewed from each site, until data saturation is reached. This is anticipated to occur within a maximum of 12 interviews. Each physiotherapist will be asked to read the clinician qualitative study PIS, and then to complete a Consent Form **(Supplementary File 2).** Physiotherapists who consent to participate will be contacted to arrange a suitable time to conduct a telephone interview.

Data collection

Interviews will be conducted four to six weeks after a participant's final physiotherapy session. This cross-sectional time point allows exploration of the participant's study

For peer review only - http://bmjopen.bmj.com/site/about/guidelines.xhtml

experience and adherence to home exercise in a reasonable recall period. Participant interviews will take up to 90 minutes. The physiotherapist interviews will take 15 to 30 minutes and will be completed within four weeks of intervention completion.

We will conduct a brief literature review of evidence into the biopsychosocial barriers and facilitators for this patient group to return to their daily activities with acceptable quality of life. In parallel, we will attend HNC patient rehabilitation groups to deepen our understanding of the patient perspective. The themes identified from the literature review and patient groups informed the semi-structured interview guide and framework. The qualitative researcher presented these to our PPI representatives and clinical experts and refined accordingly. The first iteration of the interview guide is provided in the **Supplementary File 3**. The interview schedule will be structured in alignment with the guidance for the qualitative exploration of intervention acceptability recently published in the BMJ [38]. Interviewees will have the opportunity to suggest and/or discuss additional questions. Interviews will be audio recorded, and independently transcribed.

Data analysis

Transcriptions will be managed using NVIVO software[39]. Qualitative researcher (BF) will analyse the data using framework analysis[40]. The analytical framework will be informed by our evidence synthesis of the biopsychosocial rehabilitation and behaviour change literature and refined through consultation with PPI and clinical experts. After the coding of each transcript the working framework will be discussed with patient, clinical and research team members to reduce researcher bias and strengthen the framework's reliability. The final framework will include data from participants and physiotherapists and will be triangulated

For peer review only

with quantitative data. We will produce and publish a framework of understanding for the intervention and trial progression.

Trial Status

The trial is funded for 24 months commencing in September 2019. Recruitment is expected to be complete by October 2020 with the final follow-up visit completed by April 2021. The trial will be completed by 31st August 2021. Due to the COVID-19 outbreak in the UK from March 2020, the trial timelines are expected to be extended.

Protocol changes resulting from COVID-19

The protocol was amended to reflect the NHS service delivery changes secondary to COVID-19. These amendments include allowing intervention delivery to have the option of video consultations in line with local NHS Trusts' policies. The change to online consultations has been reflected in the addition of eligbility criterion 'When the hospital is only providing video consultation physiotherapy sessions, does the patient have access to the internet through a computer or tablet'. Video-delivered interventions will be monitored via video link using NHS software. Qualitative interviews will now be conducted via telephone for both patients and physiotherapists.

Follow up data collection via telephone, and postal questionnaire data collection options have been added to minimise the need for participant hospital attendance. The study team will attempt to contact these participants on up to two occasions to remind them to complete the questionnaires. If these methods fail, we will categorise the participant as a 'non-responder' for that time-point only. Qualitative data will now be collected using telephone interviews for all groups.

For peer review only

We plan to recruit to recruit an additional 3 participants to replace the participants recruited pre-COVID who were unable to adhere to the intervention due to the emergency changes in service provision.

Patient and Public Involvement

Patient involvement began during protocol and intervention development and continues throughout the trial. A patient-member will attend all TSC meetings. The same patient-member is a co-investigator, providing insights into the trial conduct, particularly on data collection processes, and will help interpret the findings to inform on the implications of the research during the trial's dissemination phase.

ETHICS AND DISSEMINATION

Ethical approval was gained from the South Central (Oxford B) Research Ethics Committee. A TSC was appointed to independently review the data on safety, protocol adherence and recruitment to the trial. Direct access will be granted to authorised representatives from the sponsor and host institution for monitoring and/or audit of the trial to ensure compliance with regulations. Anoymised data will be shared outside the research team when required. Researchers outside the trial team may formally request for a specific data set as per the Data Management Plan. All requests will need to be approved by the TMG.

Reporting of the trial will be consistent with the CONSORT 2010 Statement and its various extensions (pilot and feasibility trials, patient reported outcomes and non-pharmcological

For peer review only - http://bmjopen.bmj.com/site/about/guidelines.xhtml

466 interventions)[41] and Template for Intervention Description and Replication (TIDieR)
467 guidelines[42]. A summary of the results and trial materials will be made available via the trial
468 website on completion of the trial. We will submit the final report to a peer-reviewed
469 academic journal.

DECLARATIONS AND ACKNOWLEDGEMENTS

Contributors: SW, TS, SL, SD researched the topic and devised the study. SW, VG, TS, SL, SD, BF, AG and RD provided the first draft of the manuscript. SD provided statistical oversight. SW, VG, TS, SL, SD, BF, AG and RD contributed equally to manuscript preparation. SW acts a guarantor. All contributors approved the final version of the manuscript.

Funding: The research is supported by the National Institute for Health Research (NIHR) Research for Patient Benefit grant (PB-PG-1217-20031). The views expressed are those of the author(s) and not necessarily those of the NHS, the NIHR or the Department of Health and Social Care.

Competing interests: None declared.

Disclaimer: None.

Ethics approval: Ethical approval was gained from the South Central (Oxford B) Research Ethics Committee (Approval Date:29 Oct 2019; Reference Number: 19/SC/0457).

For peer review only - http://bmjopen.bmj.com/site/about/guidelines.xhtml

Trial Sponsor: Oxford University Hospitals NHS Foundation Trust (OUH Research & Development, Joint Research Office, 2nd Floor, OUH Cowley, Unipart Buisness Centre, Garsington Road, Oxford, OX4 2PG. Email: OUH.Sponsorship@oxnet.nhs.uk. The views expressed are those of the author(s) and not necessarily those of the sponsor.

Provenance and peer review: Not commissioned; externally peer reviewed.

GRRAND-F Collaborators: Norfolk and Norwich University Hospitals NHS Foundation Trust, Oxford University Hospital NHS Foundation Trust, Poole Hospital NHS Foundation Trust and University Hospitals Birmingham NHS Foundation Trust and Ms Emma King, Professor Hisham Mehanna, Mr Richard Sisson, Mr Stuart Winter.

Patient consent for publication: Not required.

For peer review only

FIGURE AND TABLE LEGENDS

Box 1: Eligibility criteria

Figure 1: Study flow chart

Figure 2: GRRAND-F Intervention Schema

Table 1: Data collection schedule

Table 2: GRRAND-F objectives, outcome measures and measurement time-points

Table 3: Progression criteria for the GRRAND-F Trial.

Supplementary File 1: Participant Consent form

Supplementary File 2: Physiotherapist Consent form

Supplementary File 3: Qualitative Interview guide

For peer review only
For peer review only - http://bmjopen.bmj.com/site/about/guidelines.xhtml

REFERENCES

1. DAHNO, National Head and Neck Cancer Audit. Tenth Annual Report. 2014, The Health and Social Care Information Centre.

2. Deschler DG, et al. The "new" head and neck cancer patient-young, nonsmoker, nondrinker, and HPV positive: evaluation. Otolaryngol Head Neck Surg 2014; 151: 375-380.

3. Bray F, et al. Global estimates of cancer prevalence for 27 sites in the adult population in 2008. Int J Cancer 2013; 132: 1133-1145.

4. NCIU, Profile of Head and Neck Cancers in England: Incidence, Mortality and Survival. 2010, National Cancer Intelligence Unit: www.ncin.org.uk.

5. Schache AG, et al. HPV-related oropharyngeal cancer in the United Kingdom: an evolution in understanding of disease etiology. Cancer Res 2016; 76: 6598-6606.

6. Louie KS, et al. Trends in head and neck cancers in England from 1995 to 2011 and projections up to 2025. Oral Oncol 2015; 51: 341-348.

7. Roerink SH, et al. High prevalence of self-reported shoulder complaints after thyroid carcinoma surgery. Head & Neck 2017; 39: 260-268.

For peer review only - http://bmjopen.bmj.com/site/about/guidelines.xhtml

8. Rogers SN, et al. Quality of life following neck dissections. Acta Otolaryngol 2004; 124: 231-236.

9. Cappiello J, et al. Shoulder disability after different selective neck dissections (levels II-IV versus levels II-V): a comparative study. Laryngoscope 2005; 115: 259-263.

10. Shone GR, Yardley MP. An audit into the incidence of handicap after unilateral radical neck dissection. J Laryngol Otol 1991; 105: 760-762.

11. Cheng PT, et al. Objective comparison of shoulder dysfunction after three neck dissection techniques. Ann Otol Rhinol Laryngol 2000; 109(8 Pt 1): 761-766.

12. Chan JY, et al. Shoulder dysfunction after selective neck dissection in recurrent nasopharyngeal carcinoma. Otolaryngol Head Neck Surg, 2015; 153: 379-384.

13. Guru K, et al. A comprehensive review of head and neck cancer rehabilitation: physical therapy perspectives. Indian J Palliat Care 2012; 18: 87-97.

14. Chaplin JM, Morton RP. A prospective, longitudinal study of pain in head and neck cancer patients. Head Neck 1999; 21: 531-7.

15. NICE, Cancer of the upper aerodigestive tract: assessment and management in people aged 16 and over. [NG36]. 2016, National Institute for Health and Care Excellence.

For peer review only - http://bmjopen.bmj.com/site/about/guidelines.xhtml

16. Robinson M, et al. Provision of physiotherapy rehabilitation following neck dissection in the UK. J Laryngol Otol 2018: In Press.

17. Carvalho AP, et al. Exercise interventions for shoulder dysfunction in patients treated for head and neck cancer. Cochrane Database Syst Rev 2012; 4: CD008693.

18. Borg GA. Psychophysical bases of perceived exertion. Medicine and Science in Sports and Exercise. 1982; 14: 377-81.

19. Williamson E, Ward L, Vadher K, Dutton SJ, Parker B, Petrou S, Hutchinson CE, Gagen R, Arden NK, Barker K, Boniface G, Bruce J, Collins G, Fairbank J, Fitch J, French DP, Garrett A, Gandhi V, Griffiths F, Hansen Z, Mallen C, Morris A, Lamb SE. Better Outcomes for Older people with Spinal Trouble (BOOST) Trial: a randomised controlled trial of a combined physical and psychological intervention for older adults with neurogenic claudication, a protocol. BMJ Open. 2018 Oct 18;8(10):e022205.

20. Lamb SE, Mistry D, Alleyne S, Atherton N, Brown D, Copsey B, Dosanjh S, Finnegan S, Fordham B, Griffiths F, Hennings S, Khan I, Khan K, Lall R, Lyle S, Nichols V, Petrou S, Zeh P, Sheehan B. Aerobic and strength training exercise programme for cognitive impairment in people with mild to moderate dementia: the DAPA RCT. Health Technol Assess. 2018 May;22(28):1-202

21. International Conference on Harmonisation Good Clinical Practice (ICH GCP). International Conference on Harmonisation Good Clinical Practice Recommendations.

For peer review only

For peer review only - http://bmjopen.bmj.com/site/about/guidelines.xhtml

Accessed: 08 March 2018. Available at: https://www.ich.org/fileadmin/Public_Web_Site/ICH_Products/Guidelines/Efficacy/E6/E6_R1_Guideline.pdf

22. Roach KE, et al. Development of a shoulder pain and disability index. Arthritis Care Res 1991: 4; 143-149.

23. Eden MFA, et al. Recommendations for patient-reported outcome measures for head and neck cancer-related shoulder dysfunction: a systematic review. Rehabil Oncol 2014; 32: 6-12.

24. Dolan P, Roberts J. Modelling valuations for Eq-5d health states: an alternative model using differences in valuations. Med Care 2002; 40: 442-446.

25. Aaronson NK, et al, The European Organization for Research and Treatment of Cancer QLQC30: a quality-of-life instrument for use in international clinical trials in oncology. J Natl Cancer Inst 1993; 85: 365-376.

26. Bjordal K, et al. Quality of life in head and neck cancer patients: validation of the European Organization for Research and Treatment of Cancer Quality of Life Questionnaire-H&N35. J Clin Oncol 1999; 17: 1008-1019.

27. Singer S, Amdal CD, Hammerlid E, Tomaszewska IM, Castro Silva J, Mehanna H, et al. International validation of the revised European Organisation for Research and Treatment of Cancer Head and Neck Cancer Module, the EORTC QLQ-HN43: Phase IV. Head Neck. 2019; In Press [doi: 10.1002/hed.25609]

For peer review only - http://bmjopen.bmj.com/site/about/guidelines.xhtml

28. Teare MD, et al. Sample size requirements to estimate key design parameters from external pilot randomised controlled trials: a simulation study. Trials 2014; 15: 26

29. Baggi F, et al. Motor and functional recovery after neck dissection: comparison of two early physical rehabilitation programmes. Acta Otorhinolaryngol Ital 2014; 34: 230-240.

30. Salerno G, et al. The 11th nerve syndrome in functional neck dissection. Laryngoscope 2002; 112(7 Pt 1): 12991307.

31. Avery KNL, Williamson PR, Gamble C et al. Informing efficient randomised controlled trials: exploration of challenges in developing progression criteria for internal pilot studies. *BMJ Open* 2017; 7: e013537.

32. World Medical Association Declaration of Helsinki. World Medical Association Declaration of Helsinki (1996) and the following updates Edinburgh (2000), Seoul (2008) and Fortaleza (2013). Accessed: 08 March 2018. Available at: https://www.medunigraz.at/ethikkommission/Forum/Download/Files/DoH_1996_EN.pdf

33. Data Protection Act. UK Data Protection Act. 2018. Accessed on: 12 November 2018. Available at: https://www.gov.uk/government/collections/data-protection-act-2018

34. Lamb SE, et al. A multicentred randomised controlled trial of a primary care-based cognitive behavioural programme for low back pain. The Back Skills Training (BeST) trial. Health Technol Assess 2010; 14: 1-253.

For peer review only

35. Bruce J, Williamson E, Lait C, Richmond H, Betteley L, Lall R, Petrou S, Rees S, Withers EJ, Lamb SE, Thompson AM; PROSPER Study Group. Randomised controlled trial of exercise to prevent shoulder problems in women undergoing breast cancer treatment: study protocol for the prevention of shoulder problems trial (UK PROSPER). BMJ Open. 2018 Mar 23;8(3):e019078.

36. Lamb SE, et al, Exercises to improve function of the rheumatoid hand (SARAH): a randomised controlled trial. Lancet 2015; 385: 421-429.

37. Donovan J, Mills N, Smith M, Brindle L, Jacoby A, Peters T, Frankel S, Neal D, Hamdy F. Quality improvement report: Improving design and conduct of randomised trials by embedding them in qualitative research: ProtecT (prostate testing for cancer and treatment) study. Commentary: presenting unbiased information to patients can be difficult. BMJ. 2002;325:766-70.

38. Booth A, Noyes J, Flemming K*, et al.* Formulating questions to explore complex interventions within qualitative evidence synthesis. *BMJ Global Health* 2019;4:e001107.

39. NVivo qualitative data analysis software; QSR International Pty Ltd. Version 12, 2018.

40. Gale NK, Heath G, Cameron E, Rashid S, Redwood S. Using the framework method for the analysis of qualitative data in multi-disciplinary health research. *BMC Med Res Methodol*. 2013;13:117.

For peer review only - http://bmjopen.bmj.com/site/about/guidelines.xhtml

41. Eldridge SM, Chan CL, Campbell MJ, Bond CM, Hopewell S, Thabane L, Lancaster GA; PAFS consensus group. CONSORT 2010 statement: extension to randomised pilot and feasibility trials. BMJ. 2016; 355:i5239

42.Hoffmann TP, Glasziou PP, Boutron I, Milne R, Perera R, Moher D et al. Better reporting of interventions; template for intervention description and replication (TIDieR) checklist and guide. BMJ. 2014; 348:g1687

For peer review only

For peer review only - http://bmjopen.bmj.com/site/about/guidelines.xhtml

Box 1: GRRAND-F Eligibility Criteria

Inclusion Criteria<br>• Aged 18 years and above<br>• Being treated for HNC in whom a ND is part of their care<br>• Willing and able to provide informed consent<br>• Able to understand written English<br>• Participant is willing to attend the physiotherapy outpatient department if randomised to the experimental intervention arm (GRRAND-F intervention)<br>• Who remain eligible post-operatively when reviewed prior to randomisation
Exclusion Criteria<br>• If treatment is palliative (expected survival six months or less)<br>• Those with a pre-existing, long-term neurological disease affecting the shoulder e.g. hemiplegia<br>• Cognitive impairement (defined as an Abbreviated Mental test score of 7 or less).

For peer review only

Table 1: GRRAND-F objectives, outcome measures and measurement time-points

Objectives	Outcome Measures	Time-points
**Primary Objective**		
To determine recruitment and retention rates from study participants across sites.	Study recruitment screening logs, consent forms and logs of data collection forms completed at each time-point.	six months and 12 months (for those participants who reach this time point within the study window).
**Secondary Objectives**		
To determine potential risks of intervention contamination.	Intervention logs and qualitative interviews (face-to-face with patients/telephone-based with physiotherapists).	Completion of intervention and qualitative interviews.
To determine feasibility and acceptability of the intervention from patient and physiotherapist perspectives.	Intervention log, cross-over event as reported in protocol deviation forms, attrition rate and 'did not attend' rates for intervention. Qualitative interviews. Safety reporting forms.	Completion of intervention and qualitative interviews.
To estimate the sample size calculation for a definitive trial.	Expected primary and secondary outcome measure: Shoulder Pain and Disability Index (SPADI; overall and pain and function sub-scales); EQ-5D-5L; EORTC quality of life questionnaire (C30 core and disease-specific H&N43); health resource use questionnaire; adverse events; shoulder/neck range of motion and grip strength.	At the end of the trial.
To determine wider experiences and perceptions of the study design from a patient and physiotherapist perspective.	Qualitative interviews.	Completion of the qualitative interviews.

For peer review only - http://bmjopen.bmj.com/site/about/guidelines.xhtml

Table 2: Data collection schedule

**Data**	**Baseline**	**In-Patient Pre-Discharge**	**Intervention Period**	**6* Months Post-Randomisation**	**12* Months Post-Randomisation**
Age (years)	√				
Gender	√				
Weight (kg)/(stone/lbs)	√				
Height (cm)/(ft/inches)	√				
Ethnicity	√				
Drinking status	√				
Smoking status	√				
Primary cancer site		√			
Stage of tumour		√			
Neck nodal status		√			
Hand dominance	√				
AMTS	√				
List of medical co-morbidities	√				
Employment status and current occupation (when appropriate)	√			√	√
Shoulder Pain and Disability Index (SPADI)	√			√	√
Numerical rating scale pain	√			√	√
EQ-5D-5L	√			√	√
EORTC QLQ-C30	√			√	√
EORTC QLQ-H&HN43	√			√	√
Physical performance measures	√			√	√
Pain relief medication list	√			√	√
Complications, AE, SAE details of accident & emergency attendances and hospital admissions		√	√	√	√
Operation date		√			
Operative procedure (Level of ND)		√			
Location of HNC		√			
ASA grade		√			
Pathology results		√			
Pre-operative cancer head and neck treatment	√				
chemotherapy and radiotherapy treatment provision	√			√	√
Intervention fidelity and cross-over logs			√		
Physiotherapy intervention log (physiotherapist completed)		√	√		
Home exercise diary (participant completed)			√		
Health economic/Health utilisation questionnaire				√	√

* Each follow-up interval +/- 1 month.

For peer review only - http://bmjopen.bmj.com/site/about/guidelines.xhtml

Table 3: Progression criteria for the GRRAND-F Trial.

	Green (Go)	Amber (Amend)	Red (Stop)
**Recruitment**	60 participants recruited within 12 months	40-59 participants recruited within 12 months	<40 participants recruited within 12 months
**Consent**	≥40% of potentially eligible participants	20-39% of potentially eligible participants	<20% of potentially eligible participants
**GRRAND-F intervention fidelity**	>70% participants received protocol-compliant GRRAND-F intervention	50% to 70% received intervention as randomised	<50% received intervention as randomised
**Contamination**	<5% participants in control group received GRRAND-F intervention	5-10% participants in control group received GRRAND-F intervention	>10% participants in control group received GRRAND-F intervention
**Data Completion**	<15% missing data at 6-month follow-up	15-30% missing data	>30% missing data
**Retention**	<20% attrition at 6 month follow-up	20-50% attrition at 6 month follow-up	>50% attrition at 6 month follow-up

For peer review only

For peer review only - http://bmjopen.bmj.com/site/about/guidelines.xhtml

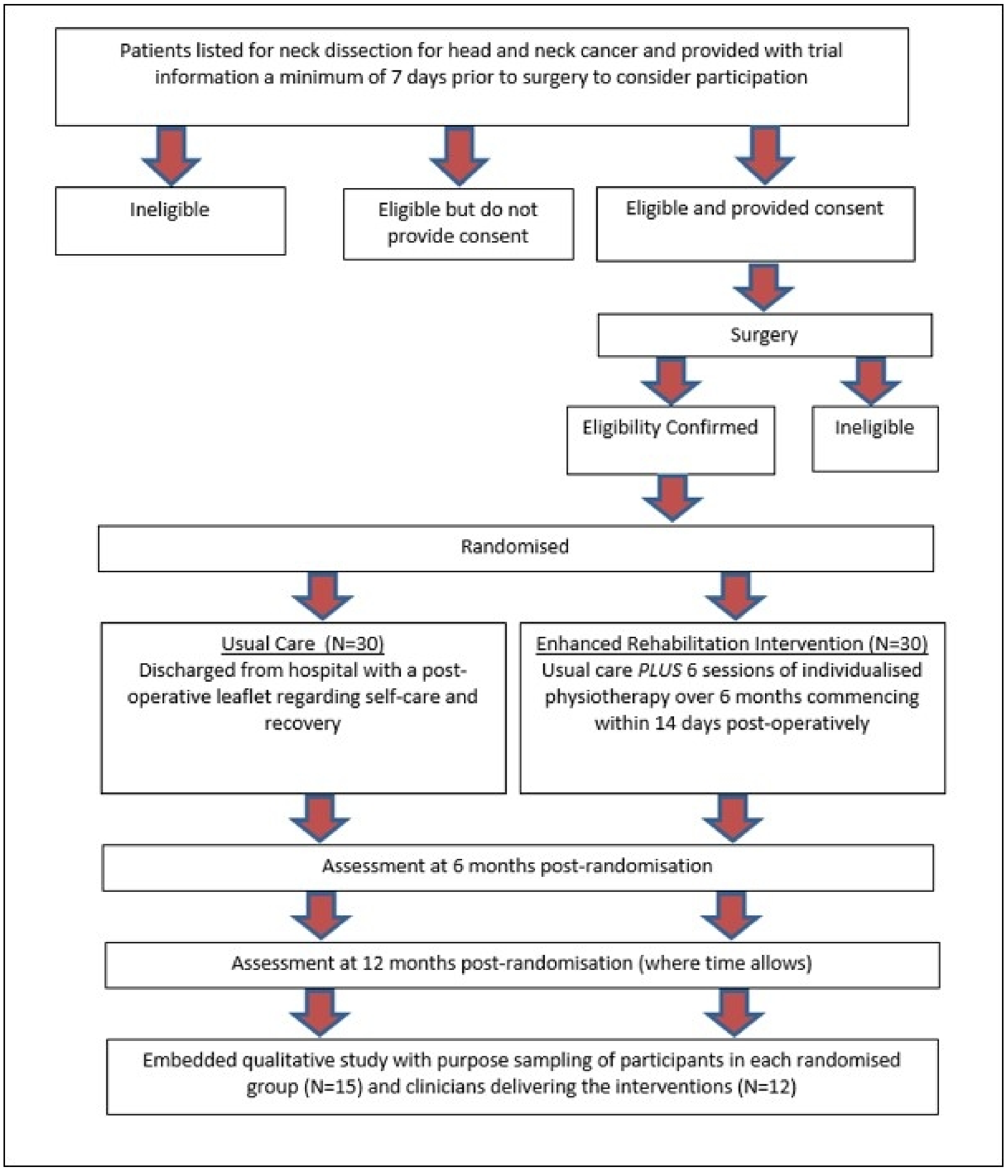

Figure 1: Study flow chart

159x185mm (96 x 96 DPI)

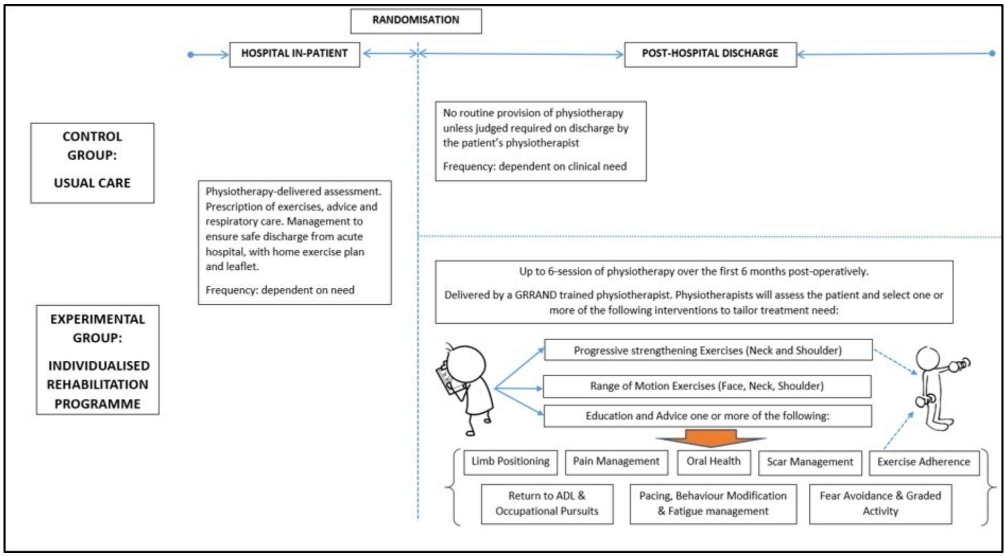

Figure 2: GRRAND-F Intervention Schema

249x137mm (96 x 96 DPI)

Sponsor Logo

CONSENT FORM (GRRAND-F STUDY)

LOCAL TRUST LOGO

Name of Local Principal Investigator:___________________________

Screening ID: | G | D | S | — | | | — | | | |

If you agree, please initial

1. I confirm that I have read and understood the Information Leaflet dated 10 June 2020 Version 4.0. I have had the opportunity to consider the information, ask questions and have had these answered satisfactorily.	
2. I understand that my participation is voluntary and that I am free to withdraw at any time without giving any reason, and without my medical care or legal rights being affected.	
3. I understand that relevant sections of my medical notes and data collected during the study may be looked at by individuals from the sponsor (XXXXXXXXXX), from regulatory authorities [and from the NHS Trust(s)], where it is relevant to me taking part in this research. I give permission for these individuals to have access to my records.	
4. I consent to the central study team holding a copy of my consent form and also my contact details so that they can contact me about the study. I understand these details will be held securely and destroyed after 5 years from the end of the study.	
5. I am aware that treatment sessions may be observed for quality assurance purposes.	
6. I agree to my General Practitioner (GP) being informed of my participation in the study.	
7. I agree to be contacted for the purposes of follow up by the central GRRAND-F team who are based in XXXXXXX.	
8. I agree to take part in the GRRAND-F study.	
**OPTIONAL**	
9. I agree to take part in the optional GRRAND-F study participant interviews.	
10. I give permission that anonymous quotes from my interview may be used in the reporting of this study.	
11. I give permission for the interview to be digitally-recorded.	
12. I agree to be contacted about ethically approved research studies for which I may be suitable. I understand that agreeing to be contacted does not oblige me to participate in any further studies.	

**Name of Participant**	**Date**	**Signature**
**Name of Person Taking Consent**	**Date**	**Signature**

SupplementaryFile1.docx IRAS ID: XXXXXXX - REC reference: XXXXXX

Original consent to be filed in site file, a copy in patient notes, a copy to participant and an electronic copy for the central study office. CI: XXXXXX

For peer review only - http://bmjopen.bmj.com/site/about/guidelines.xhtml

Sponsor Logo

CONSENT FORM (GRRAND-F STUDY) PHYSIOTHERAPIST INTERVIEW STUDY

LOCAL TRUST LOGO

Name of Local Principal Investigator: ________________________________

ID Number: G D Q - _ _ _

If you agree, please initial

1. I confirm that I have read and understood the Information Leaflet dated 10 June 2020 Version 3.0. I have had the opportunity to consider the information, ask questions and have had these answered satisfactorily.	
2. I understand that my participation is voluntary and that I am free to withdraw at any time without giving any reason, and without my legal rights being affected, any data given to the point of withdrawal would be retained.	
3. I understand that data collected during the study may be looked at by individuals from the sponsor (XXXXXXXXXXX), and from regulatory authorities, where it is relevant to me taking part in this research. I give permission for these individuals to have access to my data. I give permission for authorised individuals to have access to my data where it is relevant to this research, for a period of 5 years.	
4. I consent to the research team holding my contact details so that they can contact me about the study. I understand these details will be held securely and destroyed at the end of the study.	
5. I understand that a copy of the consent form will be kept by the local research team and a copy be sent to the central study in XXXXXXX.	
6. I give permission for anonymised written quotations from the interview to be used in reports, publications and presentations related to the study.	
7. I give permission for the interview to be digitally audio recorded.	
8. I agree to take part in this study.	

Name of Participant **Date** **Signature**

Name of Person Taking Consent **Date** **Signature**

SupplementaryFile2.docx
IRAS ID: XXXXXX - REC reference: XXXXXX CI: XXXXXXXX
Original consent to be filed in site file, a copy for physiotherapist and an electronic copy for the central study office.

For peer review only - http://bmjopen.bmj.com/site/about/guidelines.xhtml
For peer review only

GRRAND-F semi-structured interview guide Version 3.0

GRRAND-F patient

Introduction and rapport build before beginning recording. No right or wrong answers, take you time we want to learn as much as we can from you. You are the experts. Feel free to change your mind as we go along sometimes being asked different questions can make us realise we think different things. Please ask me questions before we begin or as we are chatting, this is not a formal interview it is just us talking to understand your experience. I am an independent person and my only aim to find out what is the best way we can help people rehabilitation after ND.

1. **Do you remember at what point you were approached about being part of this study?**
   a. PROBE: cancer context (diagnosis), post-operative context and now continuing with the rest of their lives context (mortality, fear, job strain etc)
   b. How were you feeling?

2. **Can you tell me what you first thought about participating in a study like this?**
   a. PROBE: positive (benefits) or negative (concerns i.e. volume of contact query)
   b. Can you recall anything that put you off agreeing to be part of the study?
   c. And / or was there anything, in particular, which made you keen to participate?

3. **When you were approached about the study, were told that you might receive one type of programme or you might receive a different type? Can you tell me about these options?**
   a. What can you remember?
   b. What did you think/feel about these options?

4. **When you were discharged from hospital, were you given a booklet of physiotherapy exercises to take home with you? Here is a copy - Show example.**
   a. Can you remember the booklet?
   b. Did this help you to perform your physiotherapy at home?
   c. Useful?
   d. Used?
   e. How could it be improved?

5. **What did you think about the physiotherapy care you received whilst you were in hospital?**

6. **You have received X (e.g. 3) sessions of physiotherapy since your operation in X (e.g. September), can you tell me what these sessions were like?**
   a. PROBE: Can you remember any specific elements which stand out to you?
   b. Parts which were very useful for you?
   c. Made a big difference in your recovery from the surgery?

For peer review only

d. How and Why?
e. Any areas which were confusing or difficult?

7. **Can you tell me, were your appointments delivered via videocalls, or face to face or a mixture of both?**
    a. What was it like for you?
    b. Can you report any problems or difficulties you had with receiving your treatment face to face or via videocall?
        i. Probe physical
            1. e.g. did you have any technical problems with the video calls?
            2. e.g. Was it ok performing the physical movements and receiving the feedback from your physio via the video calls?
        ii. Probe psychological
            1. E.g. isolation or not feeling real at home
            2. E.g. exposing and stressful at clinic
        iii. Probe social
            1. E.g. can you have time in your home to do this or does family/others breech this privacy?

8. **Were there any sessions which you were unable to attend? Can you remember why you were unable to attend? Is there anything which the physiotherapy team could have done to make it easier for you to attend?**
    a. Can you tell me about why you were not able to attend some sessions?
        i. Physical: radiotherapy/chemotherapy side-effects, pain, function, access, time?
            1. E.g. Were you feeling too tired or in pain?
        ii. Psychological: feeling low, unmotivated
            1. E.g. did they not feel that the programme was helping them?
        iii. Social: Had to look after children/work etc, radiotherapy/chemotherapy appointments?
            1. Was it the logistics?
    b. Do you think if you had received your physiotherapy sessions face to face or via videocall that this would have helped you more?
    c. Do you think anything could be changed to help with this problem?
    d. Would you have wanted more sessions?

9. **Did you think the physiotherapy sessions have helped you recover after your operation?**
    a. We aim for the rehabilitation programme to help you to do the things you want to do to and lead the life you want.
    b. Probe physical (performing exercises, movement, fatigue, functioning?)
    c. psychological (value or exercise, embarrassment of visual disfigurement, confidence)
    d. social (isolated)
        i. Why do you think it helped? What has changed? Do you think it will last? What do you think you would feel like if you had not have attended these groups?

For peer review only

ii. Why do you think it did not help? What would you suggest you should have been offered?

10. Can you identify any specific parts of the sessions which stood out for you? Parts which really helped? Parts you struggled with? And parts you did not understand why you were doing them?

a. Probe range of movement exercises (face neck and shoulder)- were these used?
   i. Probe how these helped
   ii. Swallowing
   iii. Upper limb mobility
b. Probe progressive resistance training were these used?
   i. Probe how these helped
   ii. Gradually increasing difficulty
   iii. Strength
c. Probe psychoeducation and behaviour change techniques aka what you talked about and some coping strategies which were used?
   i. Probe how these helped
   ii. Education e.g. positioning limbs, sleep, oral health, pain management, scar management,
   iii. exercise adherence - graded activities, fear avoidance, fatigue management, pacing, behaviour modification
   iv. promoting of independence and confidence

11. Did the physio give you an exercise diary and/ or a printed set of physiotherapy for you to complete at home? (show examples)

a. Can you remember what you received?
b. Was this helpful? Can you describe how you used it (if you did)?
c. Why and why not
   i. Probe capability:
      1. Physical: physically able to perform them?
      2. Psychological: did you feel that you were able to perform them?
   ii. Opportunity:
      1. Physical: Did you have space, time to perform physio exercises at home. Did you use the diary was it helpful?
      2. Social: family/friends support or not help i.e. not giving you space/time?
   iii. Motivation:
      1. Reflective: Did you think it was worth it?
      2. Automatic: worries about performing exercises?

12. You completed a set of questionnaires before and after completing the GRRAND-F programme. What did you think about these questions? (*Share the questionnaires to remind if nothing is remembered*).

a. Do they capture the issues which you think are important to you or were any issues that you think have been missed?

For peer review only

i. Probe physical, psychological and social issues

b. Were there any which you found difficult to complete?
c. Any which you did not like?
d. Were there too many or too few questionnaires?
e. Did you complete them all and if not can you explain why – could the research team change them to make them better?

13. Do you have any other feedback you would like to talk about.

a. Things which we could change in how we deliver the programme?
b. What is in the programme?
c. How many sessions you receive?
d. What happens once you have finished the programme?

For peer review only

GRRAND-F Physio

Introduction and rapport build before beginning recording. No right or wrong answers, take you time we want to learn as much as we can from you. You are the experts. Feel free to change your mind as we go along sometimes being asked different questions can make us realise we think different things. Please ask me questions before we begin or as we are chatting, this is not a formal interview it is just us talking to understand your experience. I am an independent person and our only aim to find out what is the best way we can help people rehabilitation after NC.

1. **What has it been like being part of this research study?** (Opening broad question see what is the most pertinent issues which arise)
    a. Probe differences between different sites
    b. Difficulties and benefits
    c. Things you had wished you had known before agreeing to be part of the trial?

2. **Have you worked with this patient group (i.e. HNC NC rehab) before?**
    a. Can you tell me how you felt before the study began? Any concerns?
    b. How you feel now you have been working with this group
    c. If you have been working with this groups previously, can you tell me if the patients who agreed to be part of this study were similar or different to the patients you have seen before?

3. **Can you tell me about the training you received before participating in this study?**
    a. Bests bits
    b. Bits to change
    c. Bits to add
    d. Needed more / less?

4. **After you received your training in the GRRAND-F intervention, did you think this programme would help patients?**
    a. Can you explain to me why/not?
    b. If you could change this programme what would you include/remove?
        i. Probe physical, psychological and social needs of patients

5. **Did you deliver the physiotherapy via videocalls, or face to face or a mixture of both?**
    a. What was it like for you?
    b. Barriers/problems and facilitators with either modality
        i. Probe physical (observing exercises, technical issues)
        ii. Probe psychological (connection?)
        iii. Probe social
    c. Did you have appropriate space to deliver the GRRAND-F groups either via videocalls or face to face at your place of work

6. **Did you give you patients exercise diaries to monitor the physiotherapy they did at home?**

For peer review only

a. Did you think these were useful for you to know what was going on?
b. Did you think they helped your patients?
c. Can you offer any suggestions of how to change them?

7. **Did you give your patients handouts of physiotherapy activities for them to use at home?**
    a. Were these useful?
    b. Do you think they were they used?
    c. Can you suggest any improvements?

8. **Do you think/know that your patients practiced their physiotherapy exercises between sessions? Is there anything which you can suggest that the team do to improve adherence?**
    a. Why and why not
        i. Probe capability: physical and psychological
            1. Do the patients understand and appreciate how important to their recovery it is to perform these physio exercises?
            2. Do they believe that they can perform these physio exercises?
        ii. Opportunity: probe physical and social
            1. Handouts to show them how to perform physio exercises
            2. Do they have time and support to do these rehab exercises?
        iii. Motivation: probe reflective and automatic
            1. Patients believe
            2. Patients fearful

9. **Did you experience many DNA and UTA appointments?**
    a. Were these videocall or face to face appointments?
    b. Do you remember why your patients were unable to attend?
    c. Why do you think that was?
    d. Could we do anything to change the trial or intervention to alleviate this problem?

10. **If you focus on the contents of the GRRAND-F intervention now, what do you think are the most useful elements and any suggestions for changes? Can you talk me through what you think of…**
    a. The range of movement exercises (face neck and shoulder)- were these used often, most??
        i. Probe how these helped
        ii. Swallowing
        iii. Upper limb mobility
    b. Probe progressive resistance training were these used?
        i. Probe how these helped
        ii. Gradually increasing difficulty
        iii. Strength
    c. Probe psychoeducation and behaviour change techniques aka what you talked about and some coping strategies which were used?
        i. Probe how these helped

ii. Education e.g. positioning limbs, sleep, oral health, pain management, scar management,
iii. exercise adherence - graded activities, fear avoidance, fatigue management, pacing, behaviour modification
iv. promoting of independence and confidence

11. What do you think are the major barriers to implementing an intervention such as this into usual care?
a. Workload
b. Negative consequences?
c. Could we adapt it to suit your local service needs more?

12. Do you think this programme has helped your patients?
a. We aim for the rehabilitation programme to help your patients do the things they want to do to and lead the life they want.
   i. Probe physical (performing exercises, movement, fatigue, functioning?)
   ii. psychological (value or exercise, embarrassment of visual disfigurement, confidence)
   iii. social (isolated)
b. Why do you think it helped? What has changed? Do you think it will last? What do you think they would feel like if they had not have attended these groups?
c. Why do you think it did not help? What would you suggest you should have been offered?
   i. Probe for specific ideas

13. **Do you have any other feedback you would like to talk about.**
a. Things which we could change in how we deliver the programme?
b. What is in the programme?
c. How many sessions patients receive?
d. What happens once your patients have finished the programme?
e. Or any other comments?

For peer review only

Care as usual patient

Introduction and rapport build before beginning recording. No right or wrong answers, take you time we want to learn as much as we can from you. You are the experts. Feel free to change your mind as we go along sometimes being asked different questions can make us realise we think different things. Please ask me questions before we begin or as we are chatting, this is not a formal interview it is just us talking to understand your experience. I am an independent person and our only aim to find out what is the best way we can help people rehabilitation after NC.

1. **Do you remember at what point you were approached about being part of this study?**
   a. PROBE: cancer context (diagnosis), post-operative context and now continuing with the rest of their lives context (mortality, fear, job strain etc)
   b. How were you feeling?

2. **Can you tell me what you first thought about participating in a study like this?**
   a. PROBE: positive (benefits) or negative (concerns i.e. volume of contact query)
   b. Can you recall anything that put you off agreeing to be part of the study?
   c. And / or was there anything, in particular, which made you keen to participate?

3. **When you were approached about the study were told that you might receive one type of physiotherapy or you might receive a different type. Can you tell me about these options?**
   a. What can you remember?
   b. What did you think/feel about these options?

4. **Can you tell me about the physiotherapy you received during this trial?**
   a. Was this what you were expecting?
   b. Did you hope to be in one group or another?
   c. How did you feel once you learnt what type of rehabilitation you would be receiving?

5. **When you were in hospital after your operation, do you remember the advice you received from the physiotherapist who worked with you?**
   a. What do you remember from the advice?
   b. What did you think about the advice?
   c. What would you like to change? Or stay the same?

6. **When you were discharged from hospital after your operation, did you receive a booklet of physiotherapy exercises and an exercise diary to take home with you?**
   a. Can you tell me what you thought about these?
   b. Were they useful?
   c. Have you performed any of these exercises?
   d. Do you think these should always be given out or not?

For peer review only

d. Did you complete them all and if not can you explain why – could we change them?

7. **Did you think the advice you received in hospital and the booklet you took home with you helped you with your recovery?**
    a. We aim for the rehabilitation programme to help you to do the things you want to do to and lead the life you want.
    b. Probe physical (performing exercises, movement, fatigue, functioning?)
    c. psychological (value or exercise, embarrassment of visual disfigurement, confidence)
    d. social (isolated)
        i. Why do you think it helped? What has changed? Do you think it will last?
        ii. Why do you think it did not help? What would you suggest you should have been offered?

8. **Did you perform the physiotherapy and follow the advice in the booklet**? Did you use the exercise diary?
    a. Why and why not
        iii. Probe capability: physical and psychological
        iv. Opportunity: probe physical and social
        v. Motivation: probe reflective and automatic

9. **You completed a set of questionnaires (*Share the questionnaires to remind if nothing is remembered*). What did you think about these questions?**
    a. Do they capture the issues which you think are important to you or were any issues that you think have been missed?
        vi. Probe physical, psychological and social issues
    b. Were there too many or too few questionnaires?
    c. Were there any you did not like? Did not wish to complete?

10. **Do you have any other feedback you would like to talk about.**
    a. Did you seek any other advice/help outside of the programme? Or did you feel like you needed to?
    b. Things which we could change in how we deliver the programme?
    c. What is in the programme?
    d. How many sessions you receive?
    e. What happens once you have finished the programme?
    f. Or any other comments?

For peer review only

Care as usual physio

Introduction and rapport build before beginning recording. No right or wrong answers, take you time we want to learn as much as we can from you. You are the experts. Feel free to change your mind as we go along sometimes being asked different questions can make us realise we think different things. Please ask me questions before we begin or as we are chatting, this is not a formal interview it is just us talking to understand your experience. I am an independent person and our only aim to find out what is the best way we can help people rehabilitation after NC.

1. **What has it been like being part of this research study?** (Opening broad question see what is the most pertinent issues which arise)
   a. Probe differences between different sites
   b. Difficulties and benefits
   c. Things you had wished you had known before agreeing to be part of the trial?

2. **Have you worked with this patient group (i.e. HNC NC rehab) before?**
   a. Can you tell me how you felt before the study began? Any concerns?
   b. How you feel now you have been working with this group
   c. If you have been working with this groups previously, can you tell me if the patients who agreed to be part of this study were similar or different to the patients you have seen before?

3. **Can you tell me about the training you received before participating in this study?**
   a. Bests bits
   b. Bits to change
   c. Bits to add
   d. Needed more / less?

4. **After you received your training, did you think the advice and information you were going to give to your patients would help them a lot, a little or not much?**
   a. Can you explain to me why/not?

5. **Is the advice and information you delivered to the patients very different from what you usually do with this patient group?**

6. **Did you give your patients the booklet and exercise diaries so that they could monitor their exercises at home?**
   a. Did you think the discharge booklet was useful?
   b. Did you think the exercise diary was useful?
   c. Did you think they helped your patients?
   d. Can you offer any suggestions of how to change them?

7. **Do you think the advice and information has helped your patients a lot, a little or not much?**
   a. Can you explain why or why not?

For peer review only

8. **Do you have any other feedback you would like to talk about.**
    a. Things which we could change in how we run the study?
    b. What happens once your patients have finished the programme?
    c. Or any other comments?

For peer review only

STANDARD PROTOCOL ITEMS: RECOMMENDATIONS FOR INTERVENTIONAL TRIALS

SPIRIT 2013 Checklist: Recommended items to address in a clinical trial protocol and related documents*

Section/item	ItemNo	Description	Reported on page #
**Administrative information**			
Title	1	Descriptive title identifying the study design, population, interventions, and, if applicable, trial acronym	1
Trial registration	2a	Trial identifier and registry name. If not yet registered, name of intended registry	3
	2b	All items from the World Health Organization Trial Registration Data Set	3
Protocol version	3	Date and version identifier	3
Funding	4	Sources and types of financial, material, and other support	23
Roles and responsibilities	5a	Names, affiliations, and roles of protocol contributors	1
	5b	Name and contact information for the trial sponsor	24
	5c	Role of study sponsor and funders, if any, in study design; collection, management, analysis, and interpretation of data; writing of the report; and the decision to submit the report for publication, including whether they will have ultimate authority over any of these activities	22
	5d	Composition, roles, and responsibilities of the coordinating centre, steering committee, endpoint adjudication committee, data management team, and other individuals or groups overseeing the trial, if applicable (see Item 21a for data monitoring committee)	22
**Introduction**			
Background and rationale	6a	Description of research question and justification for undertaking the trial, including summary of relevant studies (published and unpublished) examining benefits and harms for each intervention	4-6
	6b	Explanation for choice of comparators	5-6
Objectives	7	Specific objectives or hypotheses	6
Trial design	8	Description of trial design including type of trial (eg, parallel group, crossover, factorial, single group), allocation ratio, and framework (eg, superiority, equivalence, noninferiority, exploratory)	6
**Methods: Participants, interventions, and outcomes**			
Study setting	9	Description of study settings (eg, community clinic, academic hospital) and list of countries where data will be collected. Reference to where list of study sites can be obtained	7, 24
Eligibility criteria	10	Inclusion and exclusion criteria for participants. If applicable, eligibility criteria for study centres and individuals who will perform the interventions (eg, surgeons, psychotherapists)	6

For peer review only

Interventions	11a	Interventions for each group with sufficient detail to allow replication, including how and when they will be administered	8-11 (see Figure 2)
	11b	Criteria for discontinuing or modifying allocated interventions for a given trial participant (eg, drug dose change in response to harms, participant request, or improving/worsening disease)	11
	11c	Strategies to improve adherence to intervention protocols, and any procedures for monitoring adherence (eg, drug tablet return, laboratory tests)	11
	11d	Relevant concomitant care and interventions that are permitted or prohibited during the trial	12
Outcomes	12	Primary, secondary, and other outcomes, including the specific measurement variable (eg, systolic blood pressure), analysis metric (eg, change from baseline, final value, time to event), method of aggregation (eg, median, proportion), and time point for each outcome. Explanation of the clinical relevance of chosen efficacy and harm outcomes is strongly recommended	14-15 (Table 1)
Participant timeline	13	Time schedule of enrolment, interventions (including any run-ins and washouts), assessments, and visits for participants. A schematic diagram is highly recommended (see Figure)	12-14 (see Table 2 and Figure 1)
Sample size	14	Estimated number of participants needed to achieve study objectives and how it was determined, including clinical and statistical assumptions supporting any sample size calculations	15-16
Recruitment	15	Strategies for achieving adequate participant enrolment to reach target sample size	7
**Methods: Assignment of interventions (for controlled trials)**			
Allocation:			
Sequence generation	16a	Method of generating the allocation sequence (eg, computer-generated random numbers), and list of any factors for stratification. To reduce predictability of a random sequence, details of any planned restriction (eg, blocking) should be provided in a separate document that is unavailable to those who enrol participants or assign interventions	7
Allocation concealment mechanism	16b	Mechanism of implementing the allocation sequence (eg, central telephone; sequentially numbered, opaque, sealed envelopes), describing any steps to conceal the sequence until interventions are assigned	7
Implementation	16c	Who will generate the allocation sequence, who will enrol participants, and who will assign participants to interventions	7
Blinding (masking)	17a	Who will be blinded after assignment to interventions (eg, trial participants, care providers, outcome assessors, data analysts), and how	7
	17b	If blinded, circumstances under which unblinding is permissible, and procedure for revealing a participant's allocated intervention during the trial	N/A
**Methods: Data collection, management, and analysis**			
Data collection methods	18a	Plans for assessment and collection of outcome, baseline, and other trial data, including any related processes to promote data quality (eg, duplicate measurements, training of assessors) and a description of study instruments (eg, questionnaires, laboratory tests) along with their reliability and validity, if known. Reference to where data collection forms can be found, if not in the protocol	12-14, 19-20

For peer review only - http://bmjopen.bmj.com/site/about/guidelines.xhtml

	18b	Plans to promote participant retention and complete follow-up, including list of any outcome data to be collected for participants who discontinue or deviate from intervention protocols	14
Data management	19	Plans for data entry, coding, security, and storage, including any related processes to promote data quality (eg, double data entry; range checks for data values). Reference to where details of data management procedures can be found, if not in the protocol	18, 20
Statistical methods	20a	Statistical methods for analysing primary and secondary outcomes. Reference to where other details of the statistical analysis plan can be found, if not in the protocol	16-17
	20b	Methods for any additional analyses (eg, subgroup and adjusted analyses)	16-17
	20c	Definition of analysis population relating to protocol non-adherence (eg, as randomised analysis), and any statistical methods to handle missing data (eg, multiple imputation)	16-17
**Methods: Monitoring**			
Data monitoring	21a	Composition of data monitoring committee (DMC); summary of its role and reporting structure; statement of whether it is independent from the sponsor and competing interests; and reference to where further details about its charter can be found, if not in the protocol. Alternatively, an explanation of why a DMC is not needed	Feasibility Study, N/A
	21b	Description of any interim analyses and stopping guidelines, including who will have access to these interim results and make the final decision to terminate the trial	17 (See Table 3)
Harms	22	Plans for collecting, assessing, reporting, and managing solicited and spontaneously reported adverse events and other unintended effects of trial interventions or trial conduct	7
Auditing	23	Frequency and procedures for auditing trial conduct, if any, and whether the process will be independent from investigators and the sponsor	12
**Ethics and dissemination**			
Research ethics approval	24	Plans for seeking research ethics committee/institutional review board (REC/IRB) approval	N/A – Approval in place
Protocol amendments	25	Plans for communicating important protocol modifications (eg, changes to eligibility criteria, outcomes, analyses) to relevant parties (eg, investigators, REC/IRBs, trial participants, trial registries, journals, regulators)	3
Consent or assent	26a	Who will obtain informed consent or assent from potential trial participants or authorised surrogates, and how (see Item 32)	7
	26b	Additional consent provisions for collection and use of participant data and biological specimens in ancillary studies, if applicable	N/A
Confidentiality	27	How personal information about potential and enrolled participants will be collected, shared, and maintained in order to protect confidentiality before, during, and after the trial	18
Declaration of interests	28	Financial and other competing interests for principal investigators for the overall trial and each study site	23
Access to data	29	Statement of who will have access to the final trial dataset, and disclosure of contractual agreements that limit such access for investigators	22
Ancillary and post-trial care	30	Provisions, if any, for ancillary and post-trial care, and for compensation to those who suffer harm from trial participation	N/A

For peer review only - http://bmjopen.bmj.com/site/about/guidelines.xhtml

Dissemination policy	31a	Plans for investigators and sponsor to communicate trial results to participants, healthcare professionals, the public, and other relevant groups (eg, via publication, reporting in results databases, or other data sharing arrangements), including any publication restrictions	22,23
	31b	Authorship eligibility guidelines and any intended use of professional writers	1,23
	31c	Plans, if any, for granting public access to the full protocol, participant-level dataset, and statistical code	N/A
**Appendices**			
Informed consent materials	32	Model consent form and other related documentation given to participants and authorised surrogates	Supplementary File 1 and 2
Biological specimens	33	Plans for collection, laboratory evaluation, and storage of biological specimens for genetic or molecular analysis in the current trial and for future use in ancillary studies, if applicable	N/A

For peer review only

BMJ Open

Getting Recovery Right After Neck Dissection (GRRAND-F): mixed-methods Feasibility study to design a pragmatic randomised controlled trial Protocol

Journal:	*BMJ Open*
Manuscript ID	bmjopen-2020-045741.R1
Article Type:	Protocol
Date Submitted by the Author:	10-Mar-2021
Complete List of Authors:	Gallyer, Victoria; University of Oxford, Nuffield Department of Orthopaedics, Rheumatology and Musculoskeletal Sciences (NDORMS)<br>Smith, Toby O.; University of Oxford , Nuffield Department of Orthopaedics, Rheumatology and Musculoskeletal Sciences; University of East Anglia,<br>Fordham, Beth; University of Oxford , Nuffield Department of Orthopaedics, Rheumatology and Musculoskeletal Sciences (NDORMS)<br>Dutton, Susan; University of Oxford, Centre for Statistics in Medicine, Nuffield Department of Orthopaedics, Rheumatology and Musculoskeletal Sciences (NDORMS)<br>Chester-Jones, Mae; University of Oxford, Centre for Statistics in Medicine, Nuffield Department of Orthopaedics, Rheumatology and Musculoskeletal Sciences (NDORMS)<br>Lamb, Sarah; University of Exeter, College of Medicine and Health; University of Oxford, NDORMS<br>Winter, Stuart; University of Oxford, Nuffield Department of Surgical Sciences
**Primary Subject Heading**:	Surgery
Secondary Subject Heading:	Oncology, Rehabilitation medicine
Keywords:	Head & neck tumours < ONCOLOGY, Head & neck surgery < SURGERY, REHABILITATION MEDICINE

SCHOLARONE™
Manuscripts

I, the Submitting Author has the right to grant and does grant on behalf of all authors of the Work (as defined in the below author licence), an exclusive licence and/or a non-exclusive licence for contributions from authors who are: i) UK Crown employees; ii) where BMJ has agreed a CC-BY licence shall apply, and/or iii) in accordance with the terms applicable for US Federal Government officers or employees acting as part of their official duties; on a worldwide, perpetual, irrevocable, royalty-free basis to BMJ Publishing Group Ltd ("BMJ") its licensees and where the relevant Journal is co-owned by BMJ to the co-owners of the Journal, to publish the Work in this journal and any other BMJ products and to exploit all rights, as set out in our licence.

The Submitting Author accepts and understands that any supply made under these terms is made by BMJ to the Submitting Author unless you are acting as an employee on behalf of your employer or a postgraduate student of an affiliated institution which is paying any applicable article publishing charge ("APC") for Open Access articles. Where the Submitting Author wishes to make the Work available on an Open Access basis (and intends to pay the relevant APC), the terms of reuse of such Open Access shall be governed by a Creative Commons licence – details of these licences and which Creative Commons licence will apply to this Work are set out in our licence referred to above.

Other than as permitted in any relevant BMJ Author's Self Archiving Policies, I confirm this Work has not been accepted for publication elsewhere, is not being considered for publication elsewhere and does not duplicate material already published. I confirm all authors consent to publication of this Work and authorise the granting of this licence.

For peer review only

Getting Recovery Right After Neck Dissection (GRRAND-F): mixed-methods Feasibility study to design a pragmatic randomised controlled trial Protocol

Authors: Victoria H Gallyer MSc[1], Toby O Smith PhD[1,2], Beth Fordham PhD[1], Susan J Dutton MSc[3], Mae Chester-Jones MSc[3], Sarah E Lamb DPhil[1,4], Stuart C Winter MD, FRCS[5] on behalf of the GRRAND-F Trial Collaborators.

Affiliations

[1] Centre for Rehabilitation Research in Oxford, Nuffield Department of Orthopaedics, Rheumatology and Musculoskeletal Sciences (NDORMS), University of Oxford, UK

[2] Faculty of Medicine and Health Sciences, University of East Anglia, Norwich, UK

[3] Centre for Statistics in Medicine, Nuffield Department of Orthopaedics, Rheumatology and Musculoskeletal Sciences (NDORMS), University of Oxford, UK

[4] College of Medicine and Health, University of Exeter, UK

[5] Nuffield Department of Surgical Sciences, University of Oxford. Oxford University Hospitals NHS Foundation Trust, UK

Corresponding Author: Mr Stuart Winter, Department of Head and Neck Surgery, Oxford University Hospitals NHS Foundation Trust, Churchill Hospital, Headington, Oxford, UK stuart.winter@nds.ox.ac.uk. Telephone: 01865 227138, Fax: 01865 231091

Keywords: Head and neck cancer, Neck dissection, Rehabilitation, Physiotherapy

Word Count: 4662

For peer review only - http://bmjopen.bmj.com/site/about/guidelines.xhtml

ABSTRACT

INTRODUCTION: We will evaluate the feasibility of a randomised controlled trial (RCT) to estimate the effectiveness and cost-effectiveness of a rehabilitation intervention on pain, function and health-related quality of life following neck dissection (ND) after head and neck cancer (HNC).

METHODS AND ANALYSIS: This is a pragmatic, multicentred, feasibility study. Participants are randomised to usual care (control) or usual care plus an individualised, rehabilitation programme (GRRAND Intervention). Adults aged over 18 with HNC for whom neck dissection is part of their care will be recruited from specialist clinics. Participants are randomised in 1:1 ratio using a web-based service. The target sample size is 60 participants. Usual care will be received by all participants during their post-operative inpatient stay consisting standard NHS care supplemented with a booklet advising on post-operative self-management strategies. The GRRAND intervention programme consists of usual care plus up to six individual physiotherapy sessions including neck and shoulder range of motion and progressive resistance exercises, advice and education. Between sessions participants will be advised to complete a home exercise programme. The primary outcome is to determine recruitment and retention rates from study participants across sites. Outcomes will be measured at six and 12 months. Participants and physiotherapists will be invited to an optional qualitative interview at the completion of their involvement in the study. The target qualitative sample size is 15 participants and 12 physiotherapists. Interviews aim to further investigate the feasibility and acceptability of the intervention and to determine wider experiences of the study design and intervention from patient and physiotherapist perspectives.

For peer review only - http://bmjopen.bmj.com/site/about/guidelines.xhtml

ETHICS AND DISSEMINATION: Ethical approval was given on 29 October 2019 (National Research Ethics Committee Number: 19/SC/0457). Results will be reported at conferences and in peer-reviewed publications.

TRIAL ISRCTN REGISTRATION NUMBER: 11979997

STATUS: trial recruitment is ongoing and is expected to be completed by 30th Aug 2021.

Strengths and limitations of this study:

- GRRAND-F (Getting Recovery Right After Neck Dissection) is a pragmatic, multicentred, randomised control feasibility trial.
- We will evaluate whether it is feasible to run a RCT to assess the effectiveness and cost-effectiveness of a rehabilitation intervention in improving pain, function and health-related quality of life following ND after HNC.
- The primary outcome is recruitment and retention rates.
- The qualitative sub-study will explore the wider experiences and perceptions of the study design and intervention from a patient and physiotherapist perspective.

For peer review only

For peer review only - http://bmjopen.bmj.com/site/about/guidelines.xhtml

INTRODUCTION

Head and neck cancer (HNC) affects 700,000 people worldwide and over 11,000 in the UK annually[1-3]. HNC refers to neoplasms at different anatomical sites. Within the UK, tumours of the oropharynx are the most common and have seen a two-fold increase in incidence over the last 20 years, largely attributed to human papillomavirus (HPV)[4,5]. During this time there has also been a 30% increase in oral cancer[4-6]. While there has been a significant increase in HNC, prognosis and survival in the UK continues to improve[4,6]. Therefore the proportion of people living with the effects of this cancer and its treatment continues to increase.

The treatment pathway for HNC is complex, due to the varied anatomical sites of disease and the needs of the patient. Treatment for HNC requires treatment of the primary site, as well as the neck when there is spread to the lymph nodes or high probability of spread. Historically almost all patients received a neck dissection (ND). With the advent of chemo-radiotherapy as a curative treatment, less patients require a ND. However even with this approach, up to 20% of patients require a ND due to residual disease[6]. Side-effects from surgery can be significant, including swallowing problems, neck and shoulder problems, difficulties sleeping, fatigue and anxiety[7,8].

Post-operative complications are common following ND[8-11]. Early complications can include shoulder pain and infection. Late complications may not appear until three months post-treatment, and can continue to present over five years[12,13]. These complications include shoulder movement dysfunction, speech, swallowing and musculoskeletal problems such as cervical contracture and muscle wastage[12]. Psychosocial complications are also

For peer review only - http://bmjopen.bmj.com/site/about/guidelines.xhtml

For peer review only

highly prevalent post-operatively, predominantly fatigue, anxiety, depression, sleep disturbance and social isolation. Sequelae of shoulder dysfunction and psychosocial complications are strongly associated with reduced return to work, with up to 50% of patients ceasing working due to shoulder disability alone[10,14].

Rehabilitation was one of 22 key questions in the 2016 National Institute for Health and Care Excellence (NICE) Clinical Guideline[15] on the management of HNC. The guideline recommends clinicians "consider progressive resistance training for people with impaired shoulder function, as soon as possible after ND". The review noted that this evidence was from small trials with a high risk of bias. The review also highlighted a knowledge gap on how to rehabilitate HNC patients' wider side-effects. The NICE guideline concluded that a prospective randomised trial was required to understand how best to promote recovery following HNC, making this a recognised National Health Service (NHS) research priority[15].

Currently there is no national standard best practice for rehabilitation following HNC. Our study development work[16] and feedback from patient and public (PPI) representatives has shown that physiotherapy practice varies across the UK. The findings suggested that rehabilitation in the form of physiotherapy is not routinely available to patients with HNC, in either in-patient or outpatient settings[16]. When rehabilitation is offered it is often not evidence-based, and targets acute respiratory care, range of motion (ROM) exercises for the neck and shoulder, and advice on positioning of the upper limb and shoulder girdle[15]. A booklet may be provided to supplement this treatment. Outpatient treatment is minimal, and most commonly reactive, driven by patient request. Whilst trials have begun to provide indicative findings on different rehabilitation strategies for this population[17,18], the current

For peer review only - http://bmjopen.bmj.com/site/about/guidelines.xhtml

evidence-base is limited in quality and only focuses on shoulder exercises. There remains a gap in knowledge on how to rehabilitate patient's wider side-effects following surgery for HNC such as fatigue, anxiety, poor sleep and return to work. Consequently, both Cochrane[19] and NICE[15] concluded that further high-quality research is needed to determine how best to promote recovery for shoulder function, quality of life and cost-effectiveness of treatment.

This study will evaluate whether it is feasible to conduct a RCT to assess the effectiveness and cost-effectiveness of a multi-modal rehabilitation intervention in improving pain, function and health-related quality of life following ND after HNC. In addition to investigating the feasibility of an enhanced rehabilitation intervention following HNC ND, this trial will also standardise usual care.

METHODS AND ANALYSIS

Trial Design

A mixed-methods feasibility study investigating the design of a RCT to test the clinical and cost-effectiveness of usual care and an individualised, rehabilitation programme (GRRAND) compared to usual care alone in patients undergoing a ND for HNC. The study flow chart is presented as **Figure 1**. **Table 1** presents a summary of trial objectives, outcome measures and time points.

Eligibility

Participants are eligible to take part in the trial if they fulfil the eligibility criteria listed in **Box 1**. All patients having a ND regardless of other associated procedures are eligible. Head and

For peer review only - http://bmjopen.bmj.com/site/about/guidelines.xhtml

neck cancer can arise at a number of anatomical sites and a ND is often combined with additional treatment such as radiotherapy to the primary site. This reflects the expected practice in HNC treatment [15]. We will record the location of cancer, specific surgical interventions and planned additional treatments such as radiotherapy, to ascertain the profile of the recruited ND cohort. This will provide information to aid sample size calculations, stratification approaches and analysis plans for confounders/modifiers in a definitive trial.

Recruitment

Potential participants will be identified from UK NHS hospital trusts as requiring a ND as part of their treatment, and will be approached by a member of the clinical team to ask whether they would like to know more about the GRRAND-F study.

They will be asked to read the Patient information sheet (PIS) and to discuss their potential participation with anyone who they feel would provide useful advice. Potential participants will also be provided with contact information for the research team who will be able to answer any questions relating to the study. The number of patients provided with the PIS will be recorded to monitor the number of patients who are approached.

Eligible patients who agree to participate will then be asked to provide their written informed consent (**Supplementary File 1**).

Randomisation, Blinding and Allocation Concealment

For peer review only

For peer review only - http://bmjopen.bmj.com/site/about/guidelines.xhtml

Following the completion of the consent process baseline data will be collected. Participants will then be randomised once their eligibility has been confirmed post-operatively prior to hospital discharge.

Participants will be randomised in a 1:1 ratio using the centralised web-based randomisation service provided by Oxford Clinical Trials Research Unit (OCTRU). Randomisation will be undertaken using minimisation to ensure balanced allocation of participants across the two treatment groups, stratified by hospital site and spinal accessory nerve sacrifice.

The minimisation algorithm will incorporate a non-deterministic element and will be seeded using simple randomisation to prevent predictability in the early stages of the study.
Due to the nature of the intervention, participants and clinicians delivering physiotherapy will not be blinded to treatment allocation.

Intervention

Usual Care

Usual care will be received by both control and experimental intervention groups.

As part of usual care, all participants will receive the same in-patient rehabilitation programme, commencing day one post-operatively (or next physiotherapy working day), consisting of:

For peer review only

For peer review only - http://bmjopen.bmj.com/site/about/guidelines.xhtml

(1) Advice to practise simple ROM exercises for the face and neck for the purpose of preventing the onset of post-surgical contracture and optimising swallowing and shoulder movement.

(2) Respiratory care, targeting sputum clearance and breathing control.

(3) Education on body positioning to reduce pressure and pull on the shoulder girdle, oral health to reduce food pocketing in the mouth, and pain management and pacing activities to optimise levels of comfort and function.

The content, dosage and timing of in-patient physiotherapy contact will be recorded.

Reflecting usual care, on discharge participants will receive a booklet providing advice on post-operative self-management strategies including exercise, pain management, return to work and activities of daily living. This has been developed by the multidisciplinary trial team and collaborations with two of the participating NHS centres in Birmingham and Oxford to ensure that the information is standardised. Reflecting current practice, once discharged from hospital, physiotherapy will not be routinely provided to these participants.

Experimental intervention

Participants randomised to this group will receive the same in-patient rehabilitation programme as participants in the Usual Care Group *PLUS* an individualised rehabilitation programme. This will be delivered by a GRRAND-F-trained physiotherapist in an outpatient setting. In the event that the participant is still an in-patient, this will be commenced in hospital and continued, post-discharge, in an outpatient setting. The frequency to which this change of setting occurs will be recorded as part of the feasibility outcomes.

For peer review only

At the initial consultation, physiotherapists will assess the participant to identify modifiable physical and psychosocial factors associated with poor recovery following HNC surgery. These may include: muscle weakness, limited ROM, reduced sensation, pain and fear avoidance beliefs. Based on this assessment, physiotherapists will prescribe from a pre-specified range of rehabilitation options (see **Figure 2**).

Programmes will be individualised to contain one, several, or all of the treatment options, dependent on participant's needs. Participants will also be provided with a home exercise programme to supplement face-to-face sessions.

<u>Individualised Rehabilitation Options</u>

(1) ROM exercises targeting muscles and joints of the face, neck and shoulder impacted by ND. The purpose of these exercises is the prevention of post-surgical contracture, and the maintenance of swallowing and upper limb mobility.

(2) Progressive resistance exercises, targeting strengthening of the neck and shoulder. Resistance loads will initially be set at a moderate level of exertion (based on the modified Borg scale of perceived exertion [20]) to permit progression, enhance motivation and adherence, and reduce the possibility of symptom flare-up. Resistance will consist resistance bands at the shoulder and isometric resistance provided by the participant's hand for neck and temporomandibular joint exercises.

Exercises will be progressed by increasing the resistance load, speed, number of repetitions and sets or by progressing the range in which the exercise is completed and through the introduction of weight-bearing exercises through the upper limb. Additionally, the exercises will become increasingly 'task specific', targeting participant's specific functional goals.

For peer review only

For peer review only - http://bmjopen.bmj.com/site/about/guidelines.xhtml

(3) Education and advice on a number of recognised potential post-operative complications including:

- Positioning limbs to prevent joint contractures
- Oral health particularly for patients following upper cervical/head/oral surgery
- Pain management for both early and later post-operative stages through positioning, taking prescribed analgesics and pacing/behaviour modfication.
- Scar management.
- Exercise adherence and return to function with fatigue management and pacing of activities
- Promote independence and confidence to return to normal activities of daily living, work, and social pursuits.

This will be delivered through the introduction of techniques of goal setting, fear avoidance, pacing and fatigue management, behaviour modification and graded activity. This has been successfully taught and delivered by the research team in previous NIHR trials (BOOST[21], DAPA[22]), to provide a basis for this new intervention. Advice will be provided through discussion during consultations and re-enforced with worksheets designed by the multi-disciplinary trial team.

The intervention may be modified in the development phase of the trial. The intervention will be finalised prior to the main trial. If there are no substantive changes, participants will contribute to the main trial analysis.

Delivery

For peer review only

For peer review only - http://bmjopen.bmj.com/site/about/guidelines.xhtml

The experimental intervention will be delivered a maximum of six sessions over a six-month period. The design will enable assessment of how many sessions are required. The first session will aim to occur within 14 days of surgery. Reflecting normal NHS practice, the initial session will be 60 minutes, and subsequent sessions up to 45 minutes in duration. The physiotherapist, in collaboration with the participant, will agree the spacing of sessions, reflecting normal clinical practice. This spacing will allow for maximum progression of the intensity of exercise over a time period sufficient to (hypothetically) produce an improvement in outcome. Treatment options may also be added or removed at each session, in line with the participant's current treatment progress and health status.

The timing and spacing of sessions around additional treatments such as radiotherapy and chemotherapy will be determined by the participant and physiotherapist. Through this, if the participant or physiotherapist feel that the intervention is not appropriate due to radiotherapy/chemotherapy side-effects such as fatigue, pain or nausea, the GRRAND intervention will be delayed until symptoms reduce. Alternatively, if the participant and physiotherapist agree that the GRRAND intervention would be beneficial alongside such treatments, this will be permitted. This reflects the individualised nature of the intervention.

Contamination

The GRRAND-F physiotherapists who deliver the experimental intervention sessions where possible will not deliver physiotherapy to those in the control group (and vice versa). The details of the physiotherapists delivering sessions will be recorded and reviewed to monitor this risk of contamination. Due to the interventions being individualised and delivered in an

For peer review only - http://bmjopen.bmj.com/site/about/guidelines.xhtml

outpatient setting, there is a low risk of participants sharing their knowledge and experience between groups, further minimising the risk of between-group contamination.

Co-Interventions

Respecting the pragmatic nature of this study design, participants from either group will not be asked to desist from receiving any other forms of treatment during the trial or follow-up periods. If a participant receives additional treatment, the details of the treatment received and the reasons for administering will be collected.

Quality Assessment

The trial will be monitored and audited in accordance with the current approved protocol, good clinical practice[23], relevant regulations and standard operating procedures (SOPs). All designated physiotherapists who deliver usual care will be taught the standardised control intervention procedures.

Physiotherapists delivering the GRRAND intervention will attend a face-to-face training session where they will be taught the intervention and processes involved by a member of the GRRAND-F team who developed the intervention (TS, VG). Each intervention physiotherapist will be monitored during a site visit at their third/fourth session. Sessions will be monitored against the protocol to determine whether there are issues around fidelity, contamination across groups or adherence/compliance of participants. Where further training or further monitoring visits are required, these will be instigated following these visits.

For peer review only - http://bmjopen.bmj.com/site/about/guidelines.xhtml

Assessments

Data will be clinical and participant-reported and collected using questionnaires at baseline and six months post-randomisation. Data will also be collected for those participants who reach 12-month follow-up during the data collection phase. This is estimated to be applicable for up to 50% of the cohort. Data will be collected alongside routine clinical appointments at each site. A primary end-point of six-months post-randomisation was chosen to provide a signal on clinical outcomes after completing the intervention. The 12-month data provides data to assess the risk of attrition and missing data at 12 months, which will assist with the development of the definitive trial if it proves to be feasible.

Baseline Assessment

Baseline data will be collected prior to randomisation once consent has been obtained, typically during the pre-operative assessment. Data collection is described in **Table 2**.

Outcome data to be collected at each of the data collection intervals are listed below.

- Shoulder pain and function measured using the well-validated Shoulder Pain and Disability Index (SPADI)[24, 25].
- Pain measured using the SPADI 5-item Pain Sub-scale[25] and a Numerical Rating Scale.
- Function measured using the SPADI 8-item Function sub-scale[25]
- Pain medication details and usage relating to head, neck and shoulder.
- Chemotherapy and radiotherapy treatment provision.
- Health-related quality of life measured using the EQ-5D-5L score[26] and the EORTC questionnaires (C30 (core)[27] and H&N43 (head and neck specific)[28,29]).

For peer review only - http://bmjopen.bmj.com/site/about/guidelines.xhtml

- Health resource use questionnaire (collection of health resources for computation of direct medical, direct nonmedical and indirect costs); additional out-of-pocket expenses; and work absence.
- Physical performance measures including goniometer-measured shoulder and neck active ROM and hand-held dynamometer-measured grip strength will be measured by an appropriately trained member of the research team.
- Adverse events: such as prolonged delayed onset muscle soreness, swelling and wound irritation.

<u>*Follow-up procedures*</u>

Data will be collected from participants at six and 12-months (if applicable) from date of surgery with a target of +/- one month, at their routine NHS check-up appointments. If participants do not attend their follow-up appointment, they will be contacted by telephone, and, if appropriate, sent the questionnaires to complete. The study team will attempt to telephone these participants on up to two occasions. If these methods fail, we will categorise the participant as a ‘non-responder’ for that time-point only. The data collection schedule is presented in **Table 2**.

Outcome Measures

Feasibility outcome data to be collected will include:

- Screening log numbers of eligible patients, including reasons for exclusion/non-participation.
- Recruitment numbers and rate; overall and per site.

For peer review only

For peer review only - http://bmjopen.bmj.com/site/about/guidelines.xhtml

- Protocol adherence, including fidelity to control and experimental interventions using treatment logs, timing and location of intervention delivery (in particular the first session) alongside frequency of physiotherapy contact. This will assist in assessing both potential between-group contamination and intervention delivery. We will also monitor the intervention delivery as part of the Quality Assurance (QA) monitoring visits. The findings of these visits will provide data on intervention location, fidelity to the protocol, and barriers or facilitators to provision across the sites.
- Follow-up completion rate and overall study retention in each study arm for the outcome measures highlighted above.

The primary and secondary outcome measures for this trial are presented in **Table 1**.

Data Analysis

Sample Size

As this is a feasibility study which is not aimed to assess treatment effects, we have not undertaken a formal power sample size calculation.

Sixty participants will be recruited, based on Teare et al's recommendation[30] that between 50 and 70 are required when continuous scale data outcomes are to be collected. This assumes a 10% drop-out. This will also provide sufficient data to answer our feasibility objectives with 30 participants from each group recruited. Based on 2017 data from two of the participating sites, approximately 160 potentially eligible participants were identified. Based on a conservative judgement of 45% recruitment rate for this rehabilitation trial with

For peer review only

For peer review only - http://bmjopen.bmj.com/site/about/guidelines.xhtml

this cohort[19,31,32], over 60 participants could be recruited within a 12-month period. This is within the required number to conduct this study.

Statistical Analysis

Recruitment and follow-up rates are the main drivers for the feasibility design on the basis that unless reasonable rates can be achieved no formal trial will be possible. Recruitment rate will be calculated as the number of participants randomised as a proportion of eligible participants. Rates will be estimated based on data collected and a 95% confidence interval determined for these measures. The rate of incomplete information either due to drop-out to the interventions or non-completion of the outcome measures will be based on the number of participants randomised. The statistical analysis will also estimate, with 95% confidence intervals, the parameters required for a formal power calculation, particularly the standard deviation of potential outcome measures.

If the estimated recruitment and follow-up rates are such that a multicentre definitive trial is possible no formal analysis will be undertaken and data from the feasibility will be locked and carried over into the definitive trial, where funding for the definitive trial has been obtained. In this case no formal analysis of treatment efficacy will be undertaken. The definitive trial will be planned based on the data collected during this feasibility study. The mean difference, standard deviation and effect size with between-group inferential statistical analyses will be estimated to determine direction and magnitude of effect and to inform a power calculation for a definitive trial.

For peer review only - http://bmjopen.bmj.com/site/about/guidelines.xhtml

The ‘traffic light’ system will be used as a guide for progression to a definitive trial (**Table 3**)[33].If any of the criteria are not met, these will be discussed by the Trial Steering Committee (TSC) to decide if a definitive trial is feasible.

Descriptive statistics will be used to describe the demographics between the two groups. Clinical outcome data will be reported depending on the type of variable: for continuous variables the means and standard deviation in each group (or median and interquartile range if non-normally distributed) together with the unadjusted and adjusted difference in means and corresponding 95% confidence intervals with analysis of covariance, adjusting for baseline values (where appropriate) and stratification factors; for categorical variables, the number and percentage of participants in each category will be reported and unadjusted and adjusted odds ratios (for binary outcomes) together with their 95% confidence intervals will be reported.

All results will be based on the intention-to-treat population. Protocol deviations will be reported as these are an important part of the feasibility assessment when planning the definitive trial.

Health Economics

Data on health care utilisation will be collected but not analysed. To answer the feasibility questions related to the health economic perspectives, we will test the completion of the health resource use questionnaire and will present the data descriptively.

Data Management

For peer review only - http://bmjopen.bmj.com/site/about/guidelines.xhtml

All data will be processed according to the Data Protection Act 2018[23,34,35] and all documents will be stored safely in confidential conditions. Trial-specific documents, except for the signed consent form and contact details, will refer to the participant with a unique study participant number and initials only. Participant identifiable data will be stored separately from trial data.

Qualitative Investigation

The embedded qualitative study will assess the feasibility and acceptability of the experimental and control interventions from the perspectives of those delivering (physiotherapists) and receiving (participants) the interventions. The format and delivery of the qualitative interviews are based on parameters successfully implemented in previous trials conducted by the research team (BeST[36], BOOST[21], PROSPER[37], SARAH[38]), and UK trials involving cancer patients[39]. Specifically, participant opinion and experience of study recruitment, intervention content, timing, and accessibility and barriers and facilitators to adherence will be sought. Qualitative themes identified will be used to modify the content and delivery of a future definitive trial.

Recruitment

Fifteen participant interviews will be conducted, involving 10 participants from the experimental intervention group and five from the control group. Based on our previous trial work[36,38], this sample size is expected to ensure data saturation across both groups, allowing for the expected larger dataset from the experimental intervention group.

For peer review only - http://bmjopen.bmj.com/site/about/guidelines.xhtml

For peer review only

All participants will be given a brief explanation of the interviews during the initial consent process. Those willing to be interviewed will indicate permission to be contacted by the qualitative researcher on the Consent Form (**Supplementary File 1**). It will be clarified that not all willing participants may be required for the interview study.

Participants who have agreed to be contacted for the interview will be purposively sampled by the qualitative researcher to ensure the 15 interview participants are demographically representative of the full study sample. Targeted demographics include age, ethnicity, employment status, and extent of ND. We estimate that the sample will include more males than females because approximately 70% of HNC cases in the UK in males.[40] We aim to invite two males for every one female we interview. However, if we are restricted in the number of participants available for interview, we will interview as many as available. We will highlight the sex of participants as part of our interpretation of our qualitative analysis.

The qualitative researcher will telephone the sampled participants, and answer any questions they may have about taking part in the interviews. If the participant agrees to take part, a time and date convenient to the participant will be arranged for an interview. Interviews will be conducted face-to-face, and occur at a location convenient to the participant, most likely in their own home.

A minimum of one physiotherapist who delivered the experimental intervention and one physiotherapist who delivered the control intervention will be interviewed from each site, until data saturation is reached. This is anticipated to occur within a maximum of 12 interviews. Each physiotherapist will be asked to read the clinician qualitative study PIS, and

For peer review only - http://bmjopen.bmj.com/site/about/guidelines.xhtml

then to complete a Consent Form **(Supplementary File 2).** Physiotherapists who consent to participate will be contacted to arrange a suitable time to conduct a telephone interview.

Data collection

Interviews will be conducted four to six weeks after a participant's final physiotherapy session. This cross-sectional time point allows exploration of the participant's study experience and adherence to home exercise in a reasonable recall period. Participant interviews will take up to 90 minutes. The physiotherapist interviews will take 15 to 30 minutes and will be completed within four weeks of intervention completion.

We conducted a brief literature review of evidence into the biopsychosocial barriers and facilitators for this patient group to return to their daily activities with acceptable quality of life. In parallel, we attended HNC patient rehabilitation groups to deepen our understanding of the patient perspective. The themes identified from the literature review and patient groups informed the semi-structured interview guide and framework. The qualitative researcher presented these to our PPI representatives and clinical experts and refined accordingly. The refined interview guide is provided in the **Supplementary File 3**. The interview schedule will be structured in alignment with the guidance for the qualitative exploration of intervention acceptability recently published in the BMJ [41]. Interviewees will have the opportunity to suggest and/or discuss additional questions. Interviews will be audio recorded, and independently transcribed.

Data analysis

For peer review only - http://bmjopen.bmj.com/site/about/guidelines.xhtml

Transcriptions will be managed using NVIVO software[42]. Qualitative researcher (BF) will analyse the data using framework analysis[43]. The analytical framework will be informed by our evidence synthesis of the biopsychosocial rehabilitation and behaviour change literature and refined through consultation with PPI and clinical experts. After the coding of each transcript the working framework will be discussed with patient, clinical and research team members to reduce researcher bias and strengthen the framework's reliability. The final framework will include data from participants and physiotherapists and will be triangulated with quantitative data. We will produce and publish a framework of understanding for the intervention and trial progression.

Trial Status

The trial is funded for 24 months commencing in September 2019. Recruitment is expected to be complete by October 2020 with the final follow-up visit completed by April 2021. The trial will be completed by 31st August 2021. Due to the COVID-19 outbreak in the UK from March 2020, the trial timelines are expected to be extended.

Protocol changes resulting from COVID-19

The protocol was amended to reflect the NHS service delivery changes secondary to COVID-19. These amendments include allowing intervention delivery to have the option of video consultations in line with local NHS Trusts' policies. The change to online consultations has been reflected in the addition of eligibility criterion 'When the hospital is only providing video consultation physiotherapy sessions, does the patient have access to the internet through a computer or tablet'. Video-delivered interventions will be monitored via video

For peer review only

For peer review only - http://bmjopen.bmj.com/site/about/guidelines.xhtml

link using NHS software. Qualitative interviews will now be conducted via telephone for both patients and physiotherapists.

Follow up data collection via telephone, and postal questionnaire data collection options have been added to minimise the need for participant hospital attendance. The study team will attempt to contact these participants on up to two occasions to remind them to complete the questionnaires. If these methods fail, we will categorise the participant as a 'non-responder' for that time-point only. Qualitative data will now be collected using telephone interviews for all groups.

We plan to recruit to recruit an additional three participants to replace the participants recruited pre-COVID who were unable to adhere to the intervention due to the emergency changes in service provision.

For peer review

Patient and Public Involvement

Patient involvement began during protocol and intervention development and continues throughout the trial. A patient-member will attend all TSC meetings. The same patient-member is a co-investigator, providing insights into the trial conduct, particularly on data collection processes, and will help interpret the findings to inform on the implications of the research during the trial's dissemination phase.

ETHICS AND DISSEMINATION

Ethical approval was gained from the South Central (Oxford B) Research Ethics Committee. A TSC was appointed to independently review the data on safety, protocol adherence and recruitment to the trial. Direct access will be granted to authorised representatives from the

For peer review only - http://bmjopen.bmj.com/site/about/guidelines.xhtml

sponsor and host institution for monitoring and/or audit of the trial to ensure compliance with regulations. Anonymised data will be shared outside the research team when required. Researchers outside the trial team may formally request for a specific data set as per the Data Management Plan. All requests will need to be approved by the TMG.

Reporting of the trial will be consistent with the CONSORT 2010 Statement and its various extensions (pilot and feasibility trials, patient reported outcomes and non-pharmacological interventions)[44] and Template for Intervention Description and Replication (TIDieR) guidelines[45]. A summary of the results and trial materials will be made available via the trial website on completion of the trial. We will submit the final report to a peer-reviewed academic journal.

For peer review only

DECLARATIONS AND ACKNOWLEDGEMENTS

Contributors: SW, TS, SL, SD researched the topic and devised the study. SW, VG, TS, SL, SD, MC-J and BF provided the first draft of the manuscript. SD provided statistical oversight. SW, VG, TS, SL, SD, MC-J and BF contributed equally to manuscript preparation. SW acts a guarantor. All contributors approved the final version of the manuscript.

Funding: The research is supported by the National Institute for Health Research (NIHR) Research for Patient Benefit grant (PB-PG-1217-20031). The views expressed are those of the author(s) and not necessarily those of the NHS, the NIHR or the Department of Health and Social Care.

For peer review only - http://bmjopen.bmj.com/site/about/guidelines.xhtml

Competing interests: None declared.

Disclaimer: None.

Ethics approval: Ethical approval was gained from the South Central (Oxford B) Research Ethics Committee (Approval Date:29 Oct 2019; Reference Number: 19/SC/0457).

Trial Sponsor: Oxford University Hospitals NHS Foundation Trust (OUH Research & Development, Joint Research Office, 2nd Floor, OUH Cowley, Unipart Buisness Centre, Garsington Road, Oxford, OX4 2PG. Email: OUH.Sponsorship@oxnet.nhs.uk. The views expressed are those of the author(s) and not necessarily those of the sponsor.

Provenance and peer review: Not commissioned; externally peer reviewed.

GRRAND-F Collaborators: Norfolk and Norwich University Hospitals NHS Foundation Trust, Oxford University Hospital NHS Foundation Trust, Poole Hospital NHS Foundation Trust and University Hospitals Birmingham NHS Foundation Trust and Ms Emma King, Professor Hisham Mehanna, Mr Richard Sisson, Mr Stuart Winter.

Patient consent for publication: Not required.

For peer review only

FIGURE AND TABLE LEGENDS

Box 1: Eligibility criteria

Figure 1: Study flow chart

Figure 2: GRRAND-F Intervention Schema

Table 1: Data collection schedule

Table 2: GRRAND-F objectives, outcome measures and measurement time-points

Table 3: Progression criteria for the GRRAND-F Trial.

Supplementary File 1: Participant Consent form

Supplementary File 2: Physiotherapist Consent form

Supplementary File 3: Qualitative Interview guide

For peer review only

For peer review only - http://bmjopen.bmj.com/site/about/guidelines.xhtml

REFERENCES

1. DAHNO, National Head and Neck Cancer Audit. Tenth Annual Report. 2014, The Health and Social Care Information Centre.

2. Deschler DG, et al. The "new" head and neck cancer patient-young, nonsmoker, nondrinker, and HPV positive: evaluation. Otolaryngol Head Neck Surg 2014; 151: 375-380.

3. Bray F, et al. Global estimates of cancer prevalence for 27 sites in the adult population in 2008. Int J Cancer 2013; 132: 1133-1145.

4. NCIU, Profile of Head and Neck Cancers in England: Incidence, Mortality and Survival. 2010, National Cancer Intelligence Unit: www.ncin.org.uk.

5. Schache AG, et al. HPV-related oropharyngeal cancer in the United Kingdom: an evolution in understanding of disease etiology. Cancer Res 2016; 76: 6598-6606.

6. Louie KS, et al. Trends in head and neck cancers in England from 1995 to 2011 and projections up to 2025. Oral Oncol 2015; 51: 341-348.

7. Roerink SH, et al. High prevalence of self-reported shoulder complaints after thyroid carcinoma surgery. Head Neck 2017; 39: 260-268.

For peer review only

8. Rogers SN, et al. Quality of life following neck dissections. Acta Otolaryngol 2004; 124: 231-236.

9. Cappiello J, et al. Shoulder disability after different selective neck dissections (levels II-IV versus levels II-V): a comparative study. Laryngoscope 2005; 115: 259-263.

10. Shone GR, Yardley MP. An audit into the incidence of handicap after unilateral radical neck dissection. J Laryngol Otol 1991; 105: 760-762.

11. Cheng PT, et al. Objective comparison of shoulder dysfunction after three neck dissection techniques. Ann Otol Rhinol Laryngol 2000; 109(8 Pt 1): 761-766.

12. Chan JY, et al. Shoulder dysfunction after selective neck dissection in recurrent nasopharyngeal carcinoma. Otolaryngol Head Neck Surg 2015; 153: 379-384.

13. Guru K, et al. A comprehensive review of head and neck cancer rehabilitation: physical therapy perspectives. Indian J Palliat Care 2012; 18: 87-97.

14. Chaplin JM, Morton RP. A prospective, longitudinal study of pain in head and neck cancer patients. Head Neck 1999; 21: 531-7.

15. NICE, Cancer of the upper aerodigestive tract: assessment and management in people aged 16 and over. [NG36]. 2016, National Institute for Health and Care Excellence.

For peer review only

For peer review only - http://bmjopen.bmj.com/site/about/guidelines.xhtml

16. Robinson M, et al. Provision of physiotherapy rehabilitation following neck dissection in the UK. J Laryngol Otol 2018: In Press.

17. McGarvey AC, Hoffman GR, Osmotherly PG, Chiarelli PE. Maximizing shoulder function after accessory nerve injury and neck dissection surgery: A multicenter randomized controlled trial. Head Neck 2015;37:1022-31.

18. McNeely ML, Parliament MB, Seikaly H, Jha N, Magee DJ, Haykowsky MJ, Courneya KS. Effect of exercise on upper extremity pain and dysfunction in head and neck cancer survivors. Cancer 2008;113:214-22.

19. Carvalho AP, et al. Exercise interventions for shoulder dysfunction in patients treated for head and neck cancer. Cochrane Database Syst Rev 2012;4:CD008693.

20. Borg GA. Psychophysical bases of perceived exertion. Medicine Sci Sport Exercise 1982;14:377-81.

21. Williamson E, Ward L, Vadher K, Dutton SJ, Parker B, Petrou S, Hutchinson CE, Gagen R, Arden NK, Barker K, Boniface G, Bruce J, Collins G, Fairbank J, Fitch J, French DP, Garrett A, Gandhi V, Griffiths F, Hansen Z, Mallen C, Morris A, Lamb SE. Better Outcomes for Older people with Spinal Trouble (BOOST) Trial: a randomised controlled trial of a combined physical and psychological intervention for older adults with neurogenic claudication, a protocol. BMJ Open 2018;8:e022205.

For peer review only

22. Lamb SE, Mistry D, Alleyne S, Atherton N, Brown D, Copsey B, Dosanjh S, Finnegan S, Fordham B, Griffiths F, Hennings S, Khan I, Khan K, Lall R, Lyle S, Nichols V, Petrou S, Zeh P, Sheehan B. Aerobic and strength training exercise programme for cognitive impairment in people with mild to moderate dementia: the DAPA RCT. Health Technol Assess 2018;22:1-202

23. International Conference on Harmonisation Good Clinical Practice (ICH GCP). International Conference on Harmonisation Good Clinical Practice Recommendations. Accessed: 08 March 2018. Available at: https://www.ich.org/fileadmin/Public_Web_Site/ICH_Products/Guidelines/Efficacy/E6/E6_R1_Guideline.pdf

24. Roach KE, et al. Development of a shoulder pain and disability index. Arthritis Care Res 1991:4;143-149.

25. Eden MFA, et al. Recommendations for patient-reported outcome measures for head and neck cancer-related shoulder dysfunction: a systematic review. Rehabil Oncol 2014;32:6-12.

26. Dolan P, Roberts J. Modelling valuations for Eq-5d health states: an alternative model using differences in valuations. Med Care 2002;40:442-446.

27. Aaronson NK, et al, The European Organization for Research and Treatment of Cancer QLQC30: a quality-of-life instrument for use in international clinical trials in oncology. J Natl Cancer Inst 1993;85:365-376.

For peer review only

28. Bjordal K, et al. Quality of life in head and neck cancer patients: validation of the European Organization for Research and Treatment of Cancer Quality of Life Questionnaire-H&N35. J Clin Oncol 1999;17:1008-1019.

29. Singer S, Amdal CD, Hammerlid E, Tomaszewska IM, Castro Silva J, Mehanna H, et al. International validation of the revised European Organisation for Research and Treatment of Cancer Head and Neck Cancer Module, the EORTC QLQ-HN43: Phase IV. Head Neck 2019; In Press [doi: 10.1002/hed.25609]

30. Teare MD, et al. Sample size requirements to estimate key design parameters from external pilot randomised controlled trials: a simulation study. Trials 2014; 15: 26

31. Baggi F, et al. Motor and functional recovery after neck dissection: comparison of two early physical rehabilitation programmes. Acta Otorhinolaryngol Ital 2014;34:230-240.

32. Salerno G, et al. The 11th nerve syndrome in functional neck dissection. Laryngoscope 2002;112(7 Pt 1):12991307.

33. Avery KNL, Williamson PR, Gamble C et al. Informing efficient randomised controlled trials: exploration of challenges in developing progression criteria for internal pilot studies. BMJ Open 2017;7: e013537.

34. World Medical Association Declaration of Helsinki. World Medical Association

For peer review only - http://bmjopen.bmj.com/site/about/guidelines.xhtml

Declaration of Helsinki (1996) and the following updates Edinburgh (2000), Seoul (2008) and Fortaleza (2013). Accessed: 08 March 2018. Available at: https://www.medunigraz.at/ethikkommission/Forum/Download/Files/DoH_1996_EN.pdf

35. Data Protection Act. UK Data Protection Act. 2018. Accessed on: 12 November 2018. Available at: https://www.gov.uk/government/collections/data-protection-act-2018

36. Lamb SE, et al. A multicentred randomised controlled trial of a primary care-based cognitive behavioural programme for low back pain. The Back Skills Training (BeST) trial. Health Technol Assess 2010;14:1-253.

37. Bruce J, Williamson E, Lait C, Richmond H, Betteley L, Lall R, Petrou S, Rees S, Withers EJ, Lamb SE, Thompson AM; PROSPER Study Group. Randomised controlled trial of exercise to prevent shoulder problems in women undergoing breast cancer treatment: study protocol for the prevention of shoulder problems trial (UK PROSPER). BMJ Open 2018 ;8:e019078.

38. Lamb SE, et al, Exercises to improve function of the rheumatoid hand (SARAH): a randomised controlled trial. Lancet 2015;385:421-429.

39. Tataru D, Mak V, Simo R, Davies EA, Gallagher JE. Trends in the epidemiology of head and neck cancer in London. Clin Otolaryngol 2017;42:104-14.

For peer review only

For peer review only - http://bmjopen.bmj.com/site/about/guidelines.xhtml

40. Donovan J, Mills N, Smith M, Brindle L, Jacoby A, Peters T, Frankel S, Neal D, Hamdy F. Quality improvement report: Improving design and conduct of randomised trials by embedding them in qualitative research: ProtecT (prostate testing for cancer and treatment) study. Commentary: presenting unbiased information to patients can be difficult. BMJ 2002;325:766-70.

41. Booth A, Noyes J, Flemming K, *et al.* Formulating questions to explore complex interventions within qualitative evidence synthesis. BMJ Global Health 2019;4:e001107.

42. NVivo qualitative data analysis software; QSR International Pty Ltd. Version 12, 2018.

43. Gale NK, Heath G, Cameron E, Rashid S, Redwood S. Using the framework method for the analysis of qualitative data in multi-disciplinary health research. BMC Med Res Methodol 2013;13:117.

44. Eldridge SM, Chan CL, Campbell MJ, Bond CM, Hopewell S, Thabane L, Lancaster GA; PAFS consensus group. CONSORT 2010 statement: extension to randomised pilot and feasibility trials. BMJ 2016; 355:i5239

45.Hoffmann TP, Glasziou PP, Boutron I, Milne R, Perera R, Moher D et al. Better reporting of interventions; template for intervention description and replication (TIDieR) checklist and guide. BMJ 2014;348:g1687

For peer review only - http://bmjopen.bmj.com/site/about/guidelines.xhtml

Box 1: GRRAND-F Eligibility Criteria

**Inclusion Criteria**
• Aged 18 years and above<br>• Being treated for HNC in whom a ND is part of their care<br>• Willing and able to provide informed consent<br>• Able to understand written English<br>• Participant is willing to attend the physiotherapy outpatient department if randomised to the experimental intervention arm (GRRAND-F intervention)<br>• Who remain eligible post-operatively when reviewed prior to randomisation
**Exclusion Criteria**
• If treatment is palliative (expected survival six months or less)<br>• Those with a pre-existing, long-term neurological disease affecting the shoulder e.g. hemiplegia<br>• Cognitive impairement (defined as an Abbreviated Mental test score of 7 or less).

For peer review only

Table 1: GRRAND-F objectives, outcome measures and measurement time-points

Objectives	Outcome Measures	Time-points
**Primary Objective**		
To determine recruitment and retention rates from study participants across sites.	Study recruitment screening logs, consent forms and logs of data collection forms completed at each time-point.	six months and 12 months (for those participants who reach this time point within the study window).
**Secondary Objectives**		
To determine potential risks of intervention contamination.	Intervention logs and qualitative interviews (face-to-face with patients/telephone-based with physiotherapists).	Completion of intervention and qualitative interviews.
To determine feasibility and acceptability of the intervention from patient and physiotherapist perspectives.	Intervention log, cross-over event as reported in protocol deviation forms, attrition rate and 'did not attend' rates for intervention. Qualitative interviews. Safety reporting forms.	Completion of intervention and qualitative interviews.
To estimate the sample size calculation for a definitive trial.	Expected primary and secondary outcome measure: Shoulder Pain and Disability Index (SPADI; overall and pain and function sub-scales); EQ-5D-5L; EORTC quality of life questionnaire (C30 core and disease-specific H&N43); health resource use questionnaire; adverse events; shoulder/neck range of motion and grip strength.	At the end of the trial.
To determine wider experiences and perceptions of the study design from a patient and physiotherapist perspective.	Qualitative interviews.	Completion of the qualitative interviews.

For peer review only - http://bmjopen.bmj.com/site/about/guidelines.xhtml

Table 2: Data collection schedule

Data	Baseline	In-Patient Pre-Discharge	Intervention Period	6* Months Post-Randomisation	12* Months Post-Randomisation
Age (years)	√				
Gender	√				
Weight (kg)/(stone/lbs)	√				
Height (cm)/(ft/inches)	√				
Ethnicity	√				
Drinking status	√				
Smoking status	√				
Primary cancer site		√			
Stage of tumour		√			
Neck nodal status		√			
Pre-existing shoulder or neck musculoskeletal disorder	√				
Hand dominance	√				
AMTS	√				
List of medical co-morbidities	√				
Employment status and current occupation (when appropriate)	√			√	√
Shoulder Pain and Disability Index (SPADI)	√			√	√
Numerical rating scale pain	√			√	√
EQ-5D-5L	√			√	√
EORTC QLQ-C30	√			√	√
EORTC QLQ-H&HN43	√			√	√
Physical performance measures	√			√	√
Pain relief medication list	√			√	√
Complications, AE, SAE details of accident & emergency attendances and hospital admissions		√	√	√	√
Operation date		√			
Operative procedure (Level of ND)		√			
Location of HNC		√			
Accessory nerve sacrificed		√			
ASA grade		√			
Pathology results		√			
Pre-operative cancer head and neck treatment	√				
Chemotherapy and radiotherapy treatment provision	√			√	√
Intervention fidelity and cross-over logs			√		
Physiotherapy intervention log (physiotherapist completed)		√	√		
Home exercise diary (participant completed)			√		
Health economic/Health utilisation questionnaire				√	√

For peer review only - http://bmjopen.bmj.com/site/about/guidelines.xhtml

For peer review only

* Each follow-up interval +/- 1 month.

For peer review only

For peer review only - http://bmjopen.bmj.com/site/about/guidelines.xhtml

Table 3: Progression criteria for the GRRAND-F Trial.

	Green (Go)	Amber (Amend)	Red (Stop)
**Recruitment**	60 participants recruited within 12 months	40-59 participants recruited within 12 months	<40 participants recruited within 12 months
**Consent**	≥40% of potentially eligible participants	20-39% of potentially eligible participants	<20% of potentially eligible participants
**GRRAND-F intervention fidelity**	>70% participants received protocol-compliant GRRAND-F intervention	50% to 70% received intervention as randomised	<50% received intervention as randomised
**Contamination**	<5% participants in control group received GRRAND-F intervention	5-10% participants in control group received GRRAND-F intervention	>10% participants in control group received GRRAND-F intervention
**Data Completion**	<15% missing data at 6-month follow-up	15-30% missing data	>30% missing data
**Retention**	<20% attrition at 6 month follow-up	20-50% attrition at 6 month follow-up	>50% attrition at 6 month follow-up

For peer review only

For peer review only - http://bmjopen.bmj.com/site/about/guidelines.xhtml

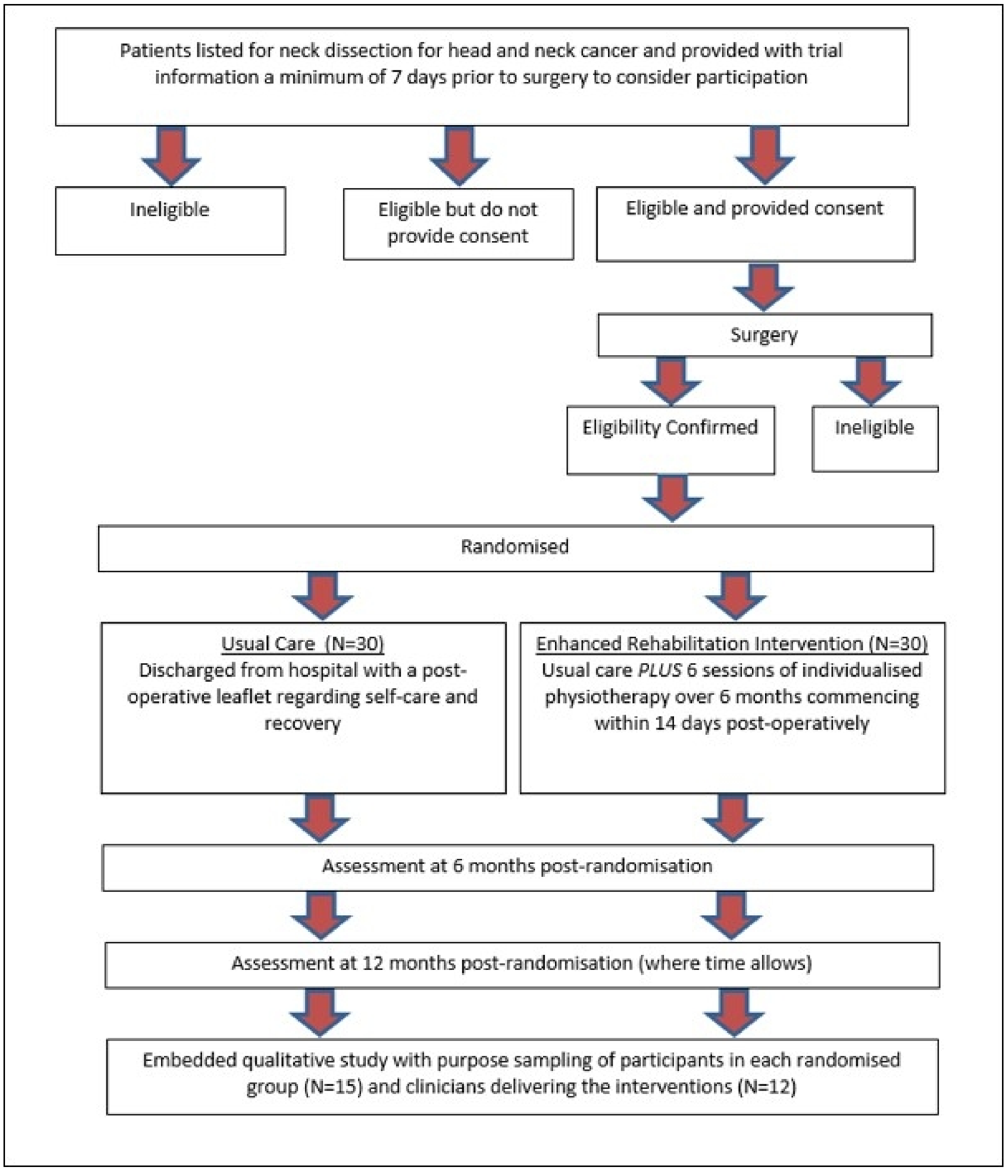

Figure 1: Study flow chart

159x185mm (96 x 96 DPI)

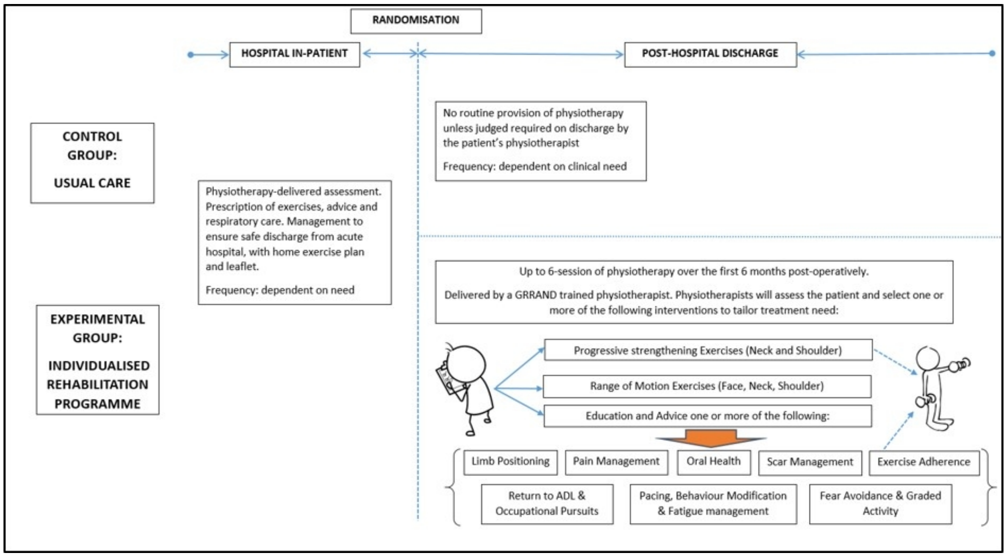

Figure 2: GRRAND-F Intervention Schema

249x137mm (96 x 96 DPI)

Sponsor Logo

CONSENT FORM (GRRAND-F STUDY)

LOCAL TRUST LOGO

Name of Local Principal Investigator:___________________________

Screening ID: | G | D | S | — | | | — | | | |

If you agree, please initial

1. I confirm that I have read and understood the Information Leaflet dated 10 June 2020 Version 4.0. I have had the opportunity to consider the information, ask questions and have had these answered satisfactorily.	
2. I understand that my participation is voluntary and that I am free to withdraw at any time without giving any reason, and without my medical care or legal rights being affected.	
3. I understand that relevant sections of my medical notes and data collected during the study may be looked at by individuals from the sponsor (XXXXXXXXXX), from regulatory authorities [and from the NHS Trust(s)], where it is relevant to me taking part in this research. I give permission for these individuals to have access to my records.	
4. I consent to the central study team holding a copy of my consent form and also my contact details so that they can contact me about the study. I understand these details will be held securely and destroyed after 5 years from the end of the study.	
5. I am aware that treatment sessions may be observed for quality assurance purposes.	
6. I agree to my General Practitioner (GP) being informed of my participation in the study.	
7. I agree to be contacted for the purposes of follow up by the central GRRAND-F team who are based in XXXXXXX.	
8. I agree to take part in the GRRAND-F study.	
**OPTIONAL**	
9. I agree to take part in the optional GRRAND-F study participant interviews.	
10. I give permission that anonymous quotes from my interview may be used in the reporting of this study.	
11. I give permission for the interview to be digitally-recorded.	
12. I agree to be contacted about ethically approved research studies for which I may be suitable. I understand that agreeing to be contacted does not oblige me to participate in any further studies.	

**Name of Participant**	**Date**	**Signature**
**Name of Person Taking Consent**	**Date**	**Signature**

SupplementaryFile1.docx IRAS ID: XXXXXXX - REC reference: XXXXXX

Original consent to be filed in site file, a copy in patient notes, a copy to participant and an electronic copy for the central study office. CI: XXXXXX

For peer review only

Sponsor Logo

GRRAND-F

CONSENT FORM (GRRAND-F STUDY) PHYSIOTHERAPIST INTERVIEW STUDY

LOCAL TRUST LOGO

Name of Local Principal Investigator: ___________________________________

ID Number: G D Q - _ _ _

If you agree, please initial

1. I confirm that I have read and understood the Information Leaflet dated 10 June 2020 Version 3.0. I have had the opportunity to consider the information, ask questions and have had these answered satisfactorily.	
2. I understand that my participation is voluntary and that I am free to withdraw at any time without giving any reason, and without my legal rights being affected, any data given to the point of withdrawal would be retained.	
3. I understand that data collected during the study may be looked at by individuals from the sponsor (XXXXXXXXXXX), and from regulatory authorities, where it is relevant to me taking part in this research. I give permission for these individuals to have access to my data. I give permission for authorised individuals to have access to my data where it is relevant to this research, for a period of 5 years.	
4. I consent to the research team holding my contact details so that they can contact me about the study. I understand these details will be held securely and destroyed at the end of the study.	
5. I understand that a copy of the consent form will be kept by the local research team and a copy be sent to the central study in XXXXXXX.	
6. I give permission for anonymised written quotations from the interview to be used in reports, publications and presentations related to the study.	
7. I give permission for the interview to be digitally audio recorded.	
8. I agree to take part in this study.	

Name of Participant **Date** **Signature**

Name of Person Taking Consent **Date** **Signature**

SupplementaryFile2.docx
IRAS ID: XXXXXX - REC reference: XXXXXX CI: XXXXXXXX
Original consent to be filed in site file, a copy for physiotherapist and an electronic copy for the central study office.

For peer review only - http://bmjopen.bmj.com/site/about/guidelines.xhtml
For peer review only

Supplementary File 3: Qualitative Interview guide

Contents

- GRRAND-F patient
- GRRAND-F physio
- Care as usual patient
- Care as usual physio

GRRAND-F patient

Introduction and rapport build before beginning recording. No right or wrong answers, take you time we want to learn as much as we can from you. You are the experts. Feel free to change your mind as we go along sometimes being asked different questions can make us realise we think different things. Please ask me questions before we begin or as we are chatting, this is not a formal interview it is just us talking to understand your experience. I am an independent person and my only aim to find out what is the best way we can help people rehabilitation after NC.

1. **Do you remember at what point you were approached about being part of this study?**
   a. PROBE: cancer context (diagnosis), post-operative context and now continuing with the rest of their lives context (mortality, fear, job strain etc)
   b. How were you feeling?

2. **Can you tell me what you first thought about participating in a study like this?**
   a. PROBE: positive (benefits) or negative (concerns i.e. volume of contact query)
   b. Can you recall anything that put you off agreeing to be part of the study?
   c. And / or was there anything, in particular, which made you keen to participate?

3. **When you were approached about the study, were told that you might receive one type of programme or you might receive a different type? Can you tell me about these options?**
   a. What can you remember?
   b. What did you think/feel about these options?

4. **When you were discharged from hospital, were you given a booklet of physiotherapy exercises to take home with you? Here is a copy - Show example.**
   a. Can you remember the booklet?
   b. Did this help you to perform your physiotherapy at home?
   c. Useful?
   d. Used?
   e. How could it be improved?

5. **What did you think about the physiotherapy care you received whilst you were in hospital?**

For peer review only

6. **You have received X (e.g. 3) sessions of physiotherapy since your operation in X (e.g. September), can you tell me what these sessions were like?**
    a. PROBE: Can you remember any specific elements which stand out to you?
    b. Parts which were very useful for you?
    c. Made a big difference in your recovery from the surgery?
    d. How and Why?
    e. Any areas which were confusing or difficult?

7. **Can you tell me, were your appointments delivered via videocalls, or face to face or a mixture of both?**
    a. What was it like for you?
    b. Can you report any problems or difficulties you had with receiving your treatment face to face or via videocall?
        i. Probe physical
            1. e.g. did you have any technical problems with the video calls?
            2. e.g. Was it ok performing the physical movements and receiving the feedback from your physio via the video calls?
        ii. Probe psychological
            1. E.g. isolation or not feeling real at home
            2. E.g. exposing and stressful at clinic
        iii. Probe social
            1. E.g. can you have time in your home to do this or does family/others breech this privacy?

8. **Were there any sessions which you were unable to attend? Can you remember why you were unable to attend? Is there anything which the physiotherapy team could have done to make it easier for you to attend?**
    a. Can you tell me about why you were not able to attend some sessions?
        i. Physical: radiotherapy/chemotherapy side-effects, pain, function, access, time?
            1. E.g. Were you feeling too tired or in pain?
        ii. Psychological: feeling low, unmotivated
            1. E.g. did they not feel that the programme was helping them?
        iii. Social: Had to look after children/work etc, radiotherapy/chemotherapy appointments?
            1. Was it the logistics?
    b. Do you think if you had received your physiotherapy sessions face to face or via videocall that this would have helped you more?
    c. Do you think anything could be changed to help with this problem?
    d. Would you have wanted more sessions?

9. **Did you think the physiotherapy sessions have helped you recover after your operation?**
    a. We aim for the rehabilitation programme to help you to do the things you want to do to and lead the life you want.
    b. Probe physical (performing exercises, movement, fatigue, functioning?)

For peer review only

c. psychological (value or exercise, embarrassment of visual disfigurement, confidence)
d. social (isolated)
    i. Why do you think it helped? What has changed? Do you think it will last? What do you think you would feel like if you had not have attended these groups?
    ii. Why do you think it did not help? What would you suggest you should have been offered?

10. Can you identify any specific parts of the sessions which stood out for you? Parts which really helped? Parts you struggled with? And parts you did not understand why you were doing them?

a. Probe range of movement exercises (face neck and shoulder)- were these used?
    i. Probe how these helped
    ii. Swallowing
    iii. Upper limb mobility
b. Probe progressive resistance training were these used?
    i. Probe how these helped
    ii. Gradually increasing difficulty
    iii. Strength
c. Probe psychoeducation and behaviour change techniques aka what you talked about and some coping strategies which were used?
    i. Probe how these helped
    ii. Education e.g. positioning limbs, sleep, oral health, pain management, scar management,
    iii. exercise adherence - graded activities, fear avoidance, fatigue management, pacing, behaviour modification
    iv. promoting of independence and confidence

11. Did the physio give you an exercise diary and/ or a printed set of physiotherapy for you to complete at home? (show examples)

a. Can you remember what you received?
b. Was this helpful? Can you describe how you used it (if you did)?
c. Why and why not
    i. Probe capability:
        1. Physical: physically able to perform them?
        2. Psychological: did you feel that you were able to perform them?
    ii. Opportunity:
        1. Physical: Did you have space, time to perform physio exercises at home. Did you use the diary was it helpful?
        2. Social: family/friends support or not help i.e. not giving you space/time?
    iii. Motivation:
        1. Reflective: Did you think it was worth it?
        2. Automatic: worries about performing exercises?

For peer review only

12. **You completed a set of questionnaires before and after completing the GRRAND-F programme. What did you think about these questions? (*Share the questionnaires to remind if nothing is remembered*).**
    a. Do they capture the issues which you think are important to you or were any issues that you think have been missed?
        i. Probe physical, psychological and social issues
    b. Were there any which you found difficult to complete?
    c. Any which you did not like?
    d. Were there too many or too few questionnaires?
    e. Did you complete them all and if not can you explain why – could the research team change them to make them better?
    f. Would you have liked to have used physical measures to test if your strength had improved?

13. **Have you sought any other type of help during your rehabilitation? outside of what we have offered you in this trial?**
    a. Paid for other therapists?
    b. Been referred within the NHS?

14. **Do you have any other feedback you would like to talk about.**
    a. Things which we could change in how we deliver the programme?
    b. What is in the programme?
    c. How many sessions you receive?
    d. What happens once you have finished the programme?

For peer review only

GRRAND-F Physio

Introduction and rapport build before beginning recording. No right or wrong answers, take you time we want to learn as much as we can from you. You are the experts. Feel free to change your mind as we go along sometimes being asked different questions can make us realise we think different things. Please ask me questions before we begin or as we are chatting, this is not a formal interview it is just us talking to understand your experience. I am an independent person and our only aim to find out what is the best way we can help people rehabilitation after NC.

1. **What has it been like being part of this research study?** (Opening broad question see what is the most pertinent issues which arise)
    a. Probe differences between different sites
    b. Difficulties and benefits
    c. Things you had wished you had known before agreeing to be part of the trial?

2. **Have you worked with this patient group (i.e. HNC NC rehab) before?**
    a. Can you tell me how you felt before the study began? Any concerns?
    b. How you feel now you have been working with this group
    c. If you have been working with this groups previously, can you tell me if the patients who agreed to be part of this study were similar or different to the patients you have seen before?

3. **Can you tell me about the training you received before participating in this study?**
    a. Bests bits
    b. Bits to change
    c. Bits to add
    d. Needed more / less?

4. **After you received your training in the GRRAND-F intervention, did you think this programme would help patients?**
    a. Can you explain to me why/not?
    b. If you could change this programme what would you include/remove?
        i. Probe physical, psychological and social needs of patients

5. **Did you deliver the physiotherapy via videocalls, or face to face or a mixture of both?**
    a. What was it like for you?
    b. Barriers/problems and facilitators with either modality
        i. Probe physical (observing exercises, technical issues)
        ii. Probe psychological (connection?)
        iii. Probe social
    c. Did you have appropriate space to deliver the GRRAND-F groups either via videocalls or face to face at your place of work

6. **Did you give you patients exercise diaries to monitor the physiotherapy they did at home?**

For peer review only

a. Did you think these were useful for you to know what was going on?
b. Did you think they helped your patients?
c. Can you offer any suggestions of how to change them?

7. **Did you give your patients handouts of physiotherapy activities for them to use at home?**
    a. Were these useful?
    b. Do you think they were they used?
    c. Can you suggest any improvements?

8. **Do you think/know that your patients practiced their physiotherapy exercises between sessions? Is there anything which you can suggest that the team do to improve adherence?**
    a. Why and why not
        i. Probe capability: physical and psychological
            1. Do the patients understand and appreciate how important to their recovery it is to perform these physio exercises?
            2. Do they believe that they can perform these physio exercises?
        ii. Opportunity: probe physical and social
            1. Handouts to show them how to perform physio exercises
            2. Do they have time and support to do these rehab exercises?
        iii. Motivation: probe reflective and automatic
            1. Patients believe
            2. Patients fearful

9. **Did you experience many DNA and UTA appointments?**
    a. Were these videocall or face to face appointments?
    b. Do you remember why your patients were unable to attend?
    c. Why do you think that was?
    d. Could we do anything to change the trial or intervention to alleviate this problem?

10. **If you focus on the contents of the GRRAND-F intervention now, what do you think are the most useful elements and any suggestions for changes? Can you talk me through what you think of…**
    a. The range of movement exercises (face neck and shoulder)- were these used often, most??
        i. Probe how these helped
        ii. Swallowing
        iii. Upper limb mobility
    b. Probe progressive resistance training were these used?
        i. Probe how these helped
        ii. Gradually increasing difficulty
        iii. Strength
    c. Probe psychoeducation and behaviour change techniques aka what you talked about and some coping strategies which were used?
        i. Probe how these helped

For peer review only

ii. Education e.g. positioning limbs, sleep, oral health, pain management, scar management,
iii. exercise adherence - graded activities, fear avoidance, fatigue management, pacing, behaviour modification
iv. promoting of independence and confidence

11. What do you think are the major barriers to implementing an intervention such as this into usual care?
   a. Workload
   b. Negative consequences?
   c. Could we adapt it to suit your local service needs more?

12. Do you think this programme has helped your patients?
   a. We aim for the rehabilitation programme to help your patients do the things they want to do to and lead the life they want.
      i. Probe physical (performing exercises, movement, fatigue, functioning?)
      ii. psychological (value or exercise, embarrassment of visual disfigurement, confidence)
      iii. social (isolated)
   b. Why do you think it helped? What has changed? Do you think it will last? What do you think they would feel like if they had not have attended these groups?
   c. Why do you think it did not help? What would you suggest you should have been offered?
      i. Probe for specific ideas

13. **Do you have any other feedback you would like to talk about.**
   a. Things which we could change in how we deliver the programme?
   b. What is in the programme?
   c. How many sessions patients receive?
   d. What happens once your patients have finished the programme?
   e. Or any other comments?

For peer review only

Care as usual patient

Introduction and rapport build before beginning recording. No right or wrong answers, take you time we want to learn as much as we can from you. You are the experts. Feel free to change your mind as we go along sometimes being asked different questions can make us realise we think different things. Please ask me questions before we begin or as we are chatting, this is not a formal interview it is just us talking to understand your experience. I am an independent person and our only aim to find out what is the best way we can help people rehabilitation after NC.

1. **Do you remember at what point you were approached about being part of this study?**
    a. PROBE: cancer context (diagnosis), post-operative context and now continuing with the rest of their lives context (mortality, fear, job strain etc)
    b. How were you feeling?

2. **Can you tell me what you first thought about participating in a study like this?**
    a. PROBE: positive (benefits) or negative (concerns i.e. volume of contact query)
    b. Can you recall anything that put you off agreeing to be part of the study?
    c. And / or was there anything, in particular, which made you keen to participate?

3. **When you were approached about the study were told that you might receive one type of physiotherapy or you might receive a different type. Can you tell me about these options?**
    a. What can you remember?
    b. What did you think/feel about these options?

4. **Can you tell me about the physiotherapy you received during this trial?**
    a. Was this what you were expecting?
    b. Did you hope to be in one group or another?
    c. How did you feel once you learnt what type of rehabilitation you would be receiving?

5. **When you were in hospital after your operation, do you remember the advice you received from the physiotherapist who worked with you?**
    a. What do you remember from the advice?
    b. What did you think about the advice?
    c. What would you like to change? Or stay the same?

6. **When you were discharged from hospital after your operation, did you receive a booklet of physiotherapy exercises and an exercise diary to take home with you?**
    a. Can you tell me what you thought about these?
    b. Were they useful?
    c. Have you performed any of these exercises?
    d. Do you think these should always be given out or not?

For peer review only

   d. Did you complete them all and if not can you explain why – could we change them?

7. **Did you think the advice you received in hospital and the booklet you took home with you helped you with your recovery?**
   a. We aim for the rehabilitation programme to help you to do the things you want to do to and lead the life you want.
   b. Probe physical (performing exercises, movement, fatigue, functioning?)
   c. psychological (value or exercise, embarrassment of visual disfigurement, confidence)
   d. social (isolated)
      i. Why do you think it helped? What has changed? Do you think it will last?
      ii. Why do you think it did not help? What would you suggest you should have been offered?

8. **Did you perform the physiotherapy and follow the advice in the booklet**? Did you use the exercise diary?
   a. Why and why not
      iii. Probe capability: physical and psychological
      iv. Opportunity: probe physical and social
      v. Motivation: probe reflective and automatic

9. **Have you sought any other therapy outside of what this trial provided to help you in your rehabilitation?**
   a. Referral within NHS
   b. Use of private services outside of NHS

10. **You completed a set of questionnaires (*Share the questionnaires to remind if nothing is remembered*). What did you think about these questions?**
   a. Do they capture the issues which you think are important to you or were any issues that you think have been missed?
      vi. Probe physical, psychological and social issues
   b. Were there too many or too few questionnaires?
   c. Were there any you did not like? Did not wish to complete?
   d. Would you expect or want am objective measurement of physical strength to see if it is changing?

11. **Do you have any other feedback you would like to talk about.**
   a. Did you seek any other advice/help outside of the programme? Or did you feel like you needed to?
   b. Things which we could change in how we deliver the programme?
   c. What is in the programme?
   d. How many sessions you receive?
   e. What happens once you have finished the programme?
   f. Or any other comments?

For peer review only

Care as usual physio

Introduction and rapport build before beginning recording. No right or wrong answers, take you time we want to learn as much as we can from you. You are the experts. Feel free to change your mind as we go along sometimes being asked different questions can make us realise we think different things. Please ask me questions before we begin or as we are chatting, this is not a formal interview it is just us talking to understand your experience. I am an independent person and our only aim to find out what is the best way we can help people rehabilitation after NC.

1. **What has it been like being part of this research study?** (Opening broad question see what is the most pertinent issues which arise)
    a. Probe differences between different sites
    b. Difficulties and benefits
    c. Things you had wished you had known before agreeing to be part of the trial?

2. **Have you worked with this patient group (i.e. HNC NC rehab) before?**
    a. Can you tell me how you felt before the study began? Any concerns?
    b. How you feel now you have been working with this group
    c. If you have been working with this groups previously, can you tell me if the patients who agreed to be part of this study were similar or different to the patients you have seen before?

3. **Can you tell me about the training you received before participating in this study?**
    a. Bests bits
    b. Bits to change
    c. Bits to add
    d. Needed more / less?

4. **After you received your training, did you think the advice and information you were going to give to your patients would help them a lot, a little or not much?**
    a. Can you explain to me why/not?

5. **Is the advice and information you delivered to the patients very different from what you usually do with this patient group?**

6. **Did you give your patients the booklet and exercise diaries so that they could monitor their exercises at home?**
    a. Did you think the discharge booklet was useful?
    b. Did you think the exercise diary was useful?
    c. Did you think they helped your patients?
    d. Can you offer any suggestions of how to change them?

7. **Do you think the advice and information has helped your patients a lot, a little or not much?**
    a. Can you explain why or why not?

For peer review only

8. **Do you have any other feedback you would like to talk about.**
    a. Things which we could change in how we run the study?
    b. What happens once your patients have finished the programme?
    c. Or any other comments?

For peer review only

SPIRIT

STANDARD PROTOCOL ITEMS: RECOMMENDATIONS FOR INTERVENTIONAL TRIALS

SPIRIT 2013 Checklist: Recommended items to address in a clinical trial protocol and related documents*

Section/item	ItemNo	Description	Reported on page #
**Administrative information**			
Title	1	Descriptive title identifying the study design, population, interventions, and, if applicable, trial acronym	1
Trial registration	2a	Trial identifier and registry name. If not yet registered, name of intended registry	3
	2b	All items from the World Health Organization Trial Registration Data Set	3
Protocol version	3	Date and version identifier	3
Funding	4	Sources and types of financial, material, and other support	23
Roles and responsibilities	5a	Names, affiliations, and roles of protocol contributors	1
	5b	Name and contact information for the trial sponsor	24
	5c	Role of study sponsor and funders, if any, in study design; collection, management, analysis, and interpretation of data; writing of the report; and the decision to submit the report for publication, including whether they will have ultimate authority over any of these activities	22
	5d	Composition, roles, and responsibilities of the coordinating centre, steering committee, endpoint adjudication committee, data management team, and other individuals or groups overseeing the trial, if applicable (see Item 21a for data monitoring committee)	22
**Introduction**			
Background and rationale	6a	Description of research question and justification for undertaking the trial, including summary of relevant studies (published and unpublished) examining benefits and harms for each intervention	4-6
	6b	Explanation for choice of comparators	5-6
Objectives	7	Specific objectives or hypotheses	6
Trial design	8	Description of trial design including type of trial (eg, parallel group, crossover, factorial, single group), allocation ratio, and framework (eg, superiority, equivalence, noninferiority, exploratory)	6
**Methods: Participants, interventions, and outcomes**			
Study setting	9	Description of study settings (eg, community clinic, academic hospital) and list of countries where data will be collected. Reference to where list of study sites can be obtained	7, 24
Eligibility criteria	10	Inclusion and exclusion criteria for participants. If applicable, eligibility criteria for study centres and individuals who will perform the interventions (eg, surgeons, psychotherapists)	6

For peer review only

Interventions	11a	Interventions for each group with sufficient detail to allow replication, including how and when they will be administered	8-11 (see Figure 2)
	11b	Criteria for discontinuing or modifying allocated interventions for a given trial participant (eg, drug dose change in response to harms, participant request, or improving/worsening disease)	11
	11c	Strategies to improve adherence to intervention protocols, and any procedures for monitoring adherence (eg, drug tablet return, laboratory tests)	11
	11d	Relevant concomitant care and interventions that are permitted or prohibited during the trial	12
Outcomes	12	Primary, secondary, and other outcomes, including the specific measurement variable (eg, systolic blood pressure), analysis metric (eg, change from baseline, final value, time to event), method of aggregation (eg, median, proportion), and time point for each outcome. Explanation of the clinical relevance of chosen efficacy and harm outcomes is strongly recommended	14-15 (Table 1)
Participant timeline	13	Time schedule of enrolment, interventions (including any run-ins and washouts), assessments, and visits for participants. A schematic diagram is highly recommended (see Figure)	12-14 (see Table 2 and Figure 1)
Sample size	14	Estimated number of participants needed to achieve study objectives and how it was determined, including clinical and statistical assumptions supporting any sample size calculations	15-16
Recruitment	15	Strategies for achieving adequate participant enrolment to reach target sample size	7
**Methods: Assignment of interventions (for controlled trials)**			
Allocation:			
Sequence generation	16a	Method of generating the allocation sequence (eg, computer-generated random numbers), and list of any factors for stratification. To reduce predictability of a random sequence, details of any planned restriction (eg, blocking) should be provided in a separate document that is unavailable to those who enrol participants or assign interventions	7
Allocation concealment mechanism	16b	Mechanism of implementing the allocation sequence (eg, central telephone; sequentially numbered, opaque, sealed envelopes), describing any steps to conceal the sequence until interventions are assigned	7
Implementation	16c	Who will generate the allocation sequence, who will enrol participants, and who will assign participants to interventions	7
Blinding (masking)	17a	Who will be blinded after assignment to interventions (eg, trial participants, care providers, outcome assessors, data analysts), and how	7
	17b	If blinded, circumstances under which unblinding is permissible, and procedure for revealing a participant's allocated intervention during the trial	N/A
**Methods: Data collection, management, and analysis**			
Data collection methods	18a	Plans for assessment and collection of outcome, baseline, and other trial data, including any related processes to promote data quality (eg, duplicate measurements, training of assessors) and a description of study instruments (eg, questionnaires, laboratory tests) along with their reliability and validity, if known. Reference to where data collection forms can be found, if not in the protocol	12-14, 19-20

	18b	Plans to promote participant retention and complete follow-up, including list of any outcome data to be collected for participants who discontinue or deviate from intervention protocols	14
Data management	19	Plans for data entry, coding, security, and storage, including any related processes to promote data quality (eg, double data entry; range checks for data values). Reference to where details of data management procedures can be found, if not in the protocol	18, 20
Statistical methods	20a	Statistical methods for analysing primary and secondary outcomes. Reference to where other details of the statistical analysis plan can be found, if not in the protocol	16-17
	20b	Methods for any additional analyses (eg, subgroup and adjusted analyses)	16-17
	20c	Definition of analysis population relating to protocol non-adherence (eg, as randomised analysis), and any statistical methods to handle missing data (eg, multiple imputation)	16-17
**Methods: Monitoring**			
Data monitoring	21a	Composition of data monitoring committee (DMC); summary of its role and reporting structure; statement of whether it is independent from the sponsor and competing interests; and reference to where further details about its charter can be found, if not in the protocol. Alternatively, an explanation of why a DMC is not needed	Feasibility Study, N/A
	21b	Description of any interim analyses and stopping guidelines, including who will have access to these interim results and make the final decision to terminate the trial	17 (See Table 3)
Harms	22	Plans for collecting, assessing, reporting, and managing solicited and spontaneously reported adverse events and other unintended effects of trial interventions or trial conduct	7
Auditing	23	Frequency and procedures for auditing trial conduct, if any, and whether the process will be independent from investigators and the sponsor	12
**Ethics and dissemination**			
Research ethics approval	24	Plans for seeking research ethics committee/institutional review board (REC/IRB) approval	N/A – Approval in place
Protocol amendments	25	Plans for communicating important protocol modifications (eg, changes to eligibility criteria, outcomes, analyses) to relevant parties (eg, investigators, REC/IRBs, trial participants, trial registries, journals, regulators)	3
Consent or assent	26a	Who will obtain informed consent or assent from potential trial participants or authorised surrogates, and how (see Item 32)	7
	26b	Additional consent provisions for collection and use of participant data and biological specimens in ancillary studies, if applicable	N/A
Confidentiality	27	How personal information about potential and enrolled participants will be collected, shared, and maintained in order to protect confidentiality before, during, and after the trial	18
Declaration of interests	28	Financial and other competing interests for principal investigators for the overall trial and each study site	23
Access to data	29	Statement of who will have access to the final trial dataset, and disclosure of contractual agreements that limit such access for investigators	22
Ancillary and post-trial care	30	Provisions, if any, for ancillary and post-trial care, and for compensation to those who suffer harm from trial participation	N/A

For peer review only - http://bmjopen.bmj.com/site/about/guidelines.xhtml

Dissemination policy	31a	Plans for investigators and sponsor to communicate trial results to participants, healthcare professionals, the public, and other relevant groups (eg, via publication, reporting in results databases, or other data sharing arrangements), including any publication restrictions	22,23
	31b	Authorship eligibility guidelines and any intended use of professional writers	1,23
	31c	Plans, if any, for granting public access to the full protocol, participant-level dataset, and statistical code	N/A
**Appendices**			
Informed consent materials	32	Model consent form and other related documentation given to participants and authorised surrogates	Supplementary File 1 and 2
Biological specimens	33	Plans for collection, laboratory evaluation, and storage of biological specimens for genetic or molecular analysis in the current trial and for future use in ancillary studies, if applicable	N/A

For peer review only
